# Design of Boron and Transition Metal Embedded Two-Dimensional Porous Carbon Nitride for Electrocatalytic Synthesis of Urea

**DOI:** 10.1021/jacs.3c12017

**Published:** 2023-12-26

**Authors:** Xin Cao, Dewei Zhang, Yongqi Gao, Oleg V. Prezhdo, Lai Xu

**Affiliations:** † Institute of Functional Nano & Soft Materials (FUNSOM), Jiangsu Key Laboratory of Advanced Negative Carbon Technologies, Jiangsu Key Laboratory for Carbon-Based Functional Materials & Devices, Joint International Research Laboratory of Carbon-Based Functional Materials and Devices, Soochow University, Suzhou, 215123, Jiangsu, People’s Republic of China; ‡ Department of Chemistry, University of Southern California, Los Angeles, California 90089, United States

## Abstract

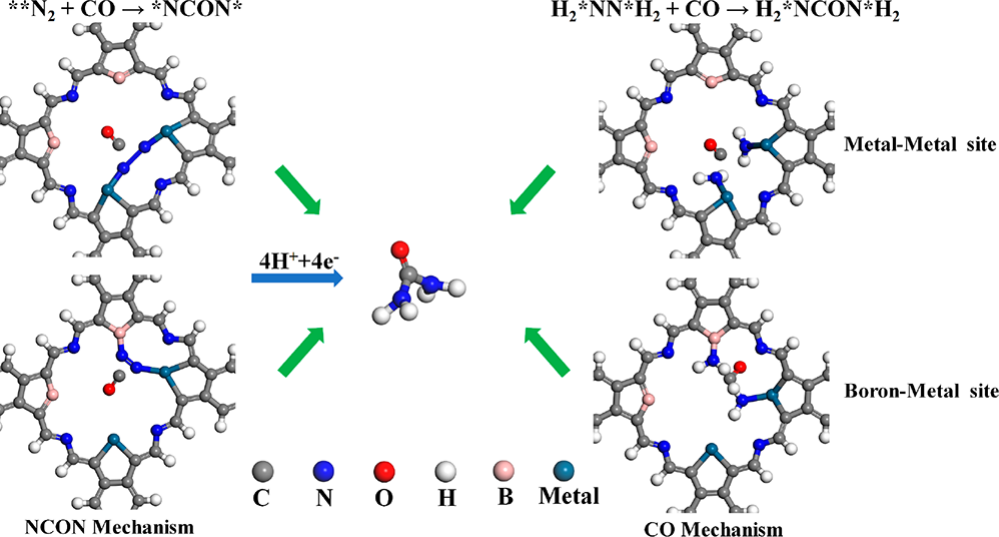

Electrocatalytic coupling of CO and N_2_ to synthesize urea under ambient conditions is considered a promising strategy to replace traditional industrial technology. It is crucial to find efficient electrocatalysts that can adsorb and activate N_2_ and promote the C–N coupling reaction. Herein, a new two-dimensional porous carbon nitride material with multiactive sites is designed, in which boron and transition metal are embedded. Through a series of screening, B_2_Cr_2_, B_2_Mn_2_, and B_2_Os_2_ are predicted to be potential electrocatalysts for urea synthesis. Mechanistic studies are performed on bidentate metal–metal and metal–boron sites, and both NCON and CO mechanisms are explored. The electronic structure analysis shows that there is a strong N_2_ chemical adsorption within the bidentate site and that the N≡N bond is significantly activated. A new mechanism where free CO is inserted for C–N coupling within the two-dimensional porous structure is proposed.

## Introduction

Urea, widely used in agriculture as a fertilizer with high nitrogen content, has potential applications in fields such as chemical industry, medicine, plastics, and textiles, and the demand for urea grows steadily.^1−3^ The traditional method of synthesizing urea involves the reaction of ammonia and carbon dioxide under high temperature and pressure:^4^ CO_2_ + 2NH_3_ → H_2_*NCON*H_2_ + H_2_O, consuming a large amount of energy and also emitting greenhouse gases, aggravating the greenhouse effect.^5,6^ Therefore, with the increasing awareness of environmental protection, there is a sharp increase in the number of clean and efficient approaches for urea production.

Electrocatalytic C–N coupling is considered as an ideal clean and efficient process to produce urea under ambient conditions,^7−11^ which could increase the added value of the product and expand the product variety. Chen et al. successfully synthesized urea via a C–N coupling reaction utilizing an electrocatalyst composed of PdCu alloy nanoparticles and titanium dioxide nanosheets with a high rate of urea formation and explored the urea formation mechanism computationally.^8^ A copper phthalocyanine nanotube is considered to be an efficient catalyst that provides multiple active sites for the co-reduction of N_2_ and CO_2_ gases, leading to the synthesis of urea with high urea yield and Faradaic efficiency.^11^ Currently, the catalytic activity and selectivity of the electrochemical synthesis of urea remain extremely low. This can be attributed to several major challenges:^12^ (1) The chemical adsorption of inert N_2_ on the catalyst surface is very weak, making it difficult to initiate the reaction. (2) The dissociation of the highly stable N≡N bond requires a high overpotential, leading to energy inefficiency. (3) The N_2_ reduction reaction competes strongly with the desired C–N coupling reaction, resulting in complex product distributions. Addressing these challenges will require innovative strategies and advanced catalyst designs to improve the catalytic activity, selectivity, and overall efficiency of electrochemical urea synthesis.

Transition metals possess empty and occupied d orbitals, which can accept and donate electrons to activate the N≡N bond.^13^ When N_2_ is adsorbed on a transition metal, the metal’s empty d orbitals can accept the lone pair electrons from N_2_, while the occupied d orbitals can donate electrons to the antibonding orbitals of N_2_, enhancing the chemical bond between the transition metal and nitrogen atom, activating the N≡N bond, and making the bond dissociation easier.^14^ Single-atom catalysts (SACs)^15−18^ embedded with metal have advantages such as clear catalytic sites, maximum atomic utilization, and excellent catalytic efficiency. Urea synthesis involves complex multistep reactions.^19−24^ A dual-atom catalyst with more flexible active sites and cooperative interatomic interactions is a promising solution to realize the effect of the cooperative sites.^25−27^ Bimetallic catalysts exhibit better orbital matching, which enhances the interaction between catalytic sites and N_2_ adsorbed with the side-on pattern, thus facilitating activation of the N≡N bond. Theoretical studies have confirmed that the side-on adsorption mode of N_2_ can achieve better orbital overlap with bimetallic catalyst sites, promoting the catalytic activity.^28^ Side-on adsorption promotes the feedback of electrons from the occupied d orbitals of metal atoms to the π* orbitals of N_2_, making it feasible to activate and dissociate the N≡N bond.^28^

In recent years, boron atoms, which possess both empty and occupied p orbitals, have been noted to exhibit properties similar to transition metals.^14,29,30^ Therefore, we designed a new two-dimensional bidentate-site structure containing metal and boron atoms for the coupling of N_2_ and CO into urea.

The two-dimensional porous structure^31−34^ combines the excellent electronic properties and high exposed atomic percentage of two-dimensional materials, as well as the high specific surface area and more active sites of porous materials.^35^ Carbon nitride provides an ideal framework for the design of efficient electrocatalysts. It exhibits high electron affinity and rich cavities and effectively stabilizes transition metals.^36^ Therefore, the two-dimensional carbon nitride porous structure is a very promising catalytic material with many superior properties. Current research of urea synthesis mainly focuses on the catalyst surfaces, and there is still limited investigation of the mechanism of coupling of CO and N_2_ into urea within two-dimensional pores. Herein, we designed a new two-dimensional porous carbon nitride structure (N_4_B_4_) as the framework for urea synthesis. We also proposed a new pore-based reaction mechanism within the two-dimensional bidentate-site porous structure: the porous structure of the new metal-embedded two-dimensional carbon–nitrogen material provides space that facilitates the insertion of free CO through an ER (Eley–Rideal) mechanism.^37^

The newly designed two-dimensional N_4_B_4_ structure exhibits a small band gap of 0.30 eV near the Fermi level. The −C–N–C– linkages expand the surface area of the pores, ensuring sufficient space for the urea synthesis reaction. The dynamic stability of the material was demonstrated by phonon spectroscopy calculations. Then N_4_B_4_ has been substitution doped by two metal atoms to form B_2_M_2_ which are conductors to improve the catalytic performance. Excellent conductivity is crucial for electrocatalysts.^38^ By embedding transition metal atoms, the conductivity of these materials can be improved. Fourteen transition metal elements were selected for substitution doping of B atoms to form B_2_M_2_ structures to enhance the conductivity. Simultaneously, B_2_M_2_ can maintain the pristine shape of the crystal cell, and it allows one to explore the performance of synthesizing urea using boron–boron, metal–boron, and metal–metal bidentate sites. Through high-throughput screening, we identified B_2_Cr_2_, B_2_Mn_2_, and B_2_Os_2_ as suitable catalysts for urea synthesis, which are all conductors. Subsequently, we verified that B–Mn sites on the B_2_Mn_2_ catalyst made the best activation effect on N_2_, resulting in optimal catalytic performance for urea synthesis with a limiting potential of −0.34 V.

## Computational Methods

Spin-polarized density functional theory was implemented using the Vienna Ab-initio Simulation Package (VASP).^39^ The ion–electron interaction was expressed by the projected augmented wave (PAW) pseudopotential.^40^ The electron exchange–correlation used the Perdew–Burke–Ernzerhof (PBE) functional in the generalized gradient approximation (GGA),^41^ taking into account van der Waals interactions in the DFT-D3 empirical dispersion correction scheme.^42^ The plane-wave cutoff energy was set to 450 eV, with an energy convergence criterion of 1 × 10^–5^ eV and a force convergence criterion of −0.05 eV/Å. Geometric structure optimization was performed using 2 × 2 × 1 k-point meshes. The VASPsol module was used to correct for the implicit solvation effects in water solutions.^43^ Phonon spectroscopy was calculated in the PHONOPY program.^44^ A vacuum region of 20 Å was added in the vertical direction to avoid interference from the interactions between periodic images. The transition state energy barrier was calculated using the climbing image nudged elastic band (CI-NEB) method.^45^ The formula for calculating the Gibbs free energy change (Δ*G*) of each basic step of urea synthesis was computed as follows:
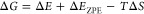
1Δ*E* is the difference of total electron energy after the reaction, and Δ*E*_ZPE_ and Δ*S* are the change of zero energy and entropy after the reaction, respectively. Δ*E*_ZPE_ and Δ*S* were obtained from the vibration analysis. We set *T* to room temperature, 298.15 K.

To demonstrate the thermodynamic stability of the B_2_M_2_ monolayer, the cohesive energy (*E*_f_) was defined as
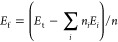
2where *E*_t_ is the total energy of the system and *n*_*i*_ and *E*_*i*_ are the number and corresponding energy of the isolated atom *i* (*i* = C, B, N, H, and metal atom) in the unit cell (*T* = 0 K). *n* = ∑_*i*_*n*_*i*_ represents the total number of atoms in the system.

The charge density difference (Δρ) was calculated as
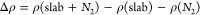
3where ρ(slab + *N*_2_) is the charge density of N_2_ adsorbed on the substrate and ρ(slab) and ρ(*N*_2_) are the charge densities of the substrate before N_2_ adsorption and the free N_2_ molecule, respectively.

## Results and Discussions

**Figure 1:**
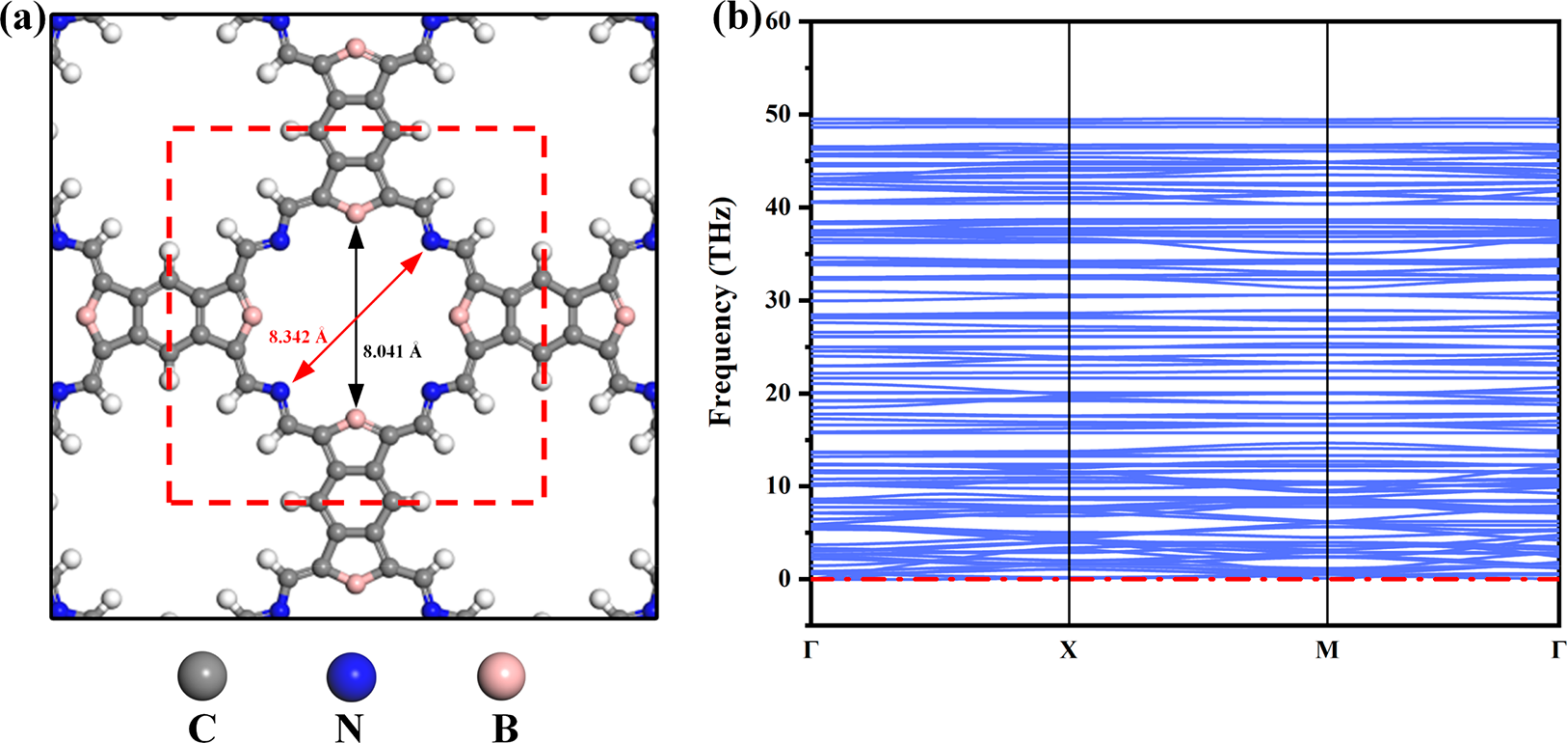
Structure and stability of N_4_B_4_. (a) Top view of the N_4_B_4_ structure and (b) phonon dispersion of N_4_B_4_ monolayers.

We first constructed four B-heterocyclic compounds by combining −C–N–C– bonds, forming a two-dimensional porous material containing four B atoms, named N_4_B_4_, as shown in Figure 1a. The unit cell of N_4_B_4_ consists of 28 C atoms, 12 H atoms, 4 N atoms, and 4 B atoms. Optimized N_4_B_4_ is stable in the P4/MMM space group, with lattice parameters of *a* = 14.494 Å, *b* = 14.494 Å, and γ = 90°. To demonstrate the dynamic stability of the structure, a phonon spectrum calculation was performed for N_4_B_4_. The phonon dispersion curves of N_4_B_4_ are all positive, without any imaginary frequencies below the 0 scale, as show in Figure 1b, illustrating the N_4_B_4_ structure is dynamically stable. Furthermore, the electronic band structures and the projected density of states (pDOS) of N_4_B_4_ were calculated. As shown in Figure S1, there is a band gap of 0.30 eV near the Fermi level. Fourteen transition metal elements (V, Cr, Mn, Fe, Co, Ni, Cu, Zn, Mo, Rh, Ru, Pd, Os, and Ir) were chosen to selectively dope on N_4_B_4_ to replace B atoms and enhance the conductivity of the structure, while also serving as active sites for urea synthesis, and finally the changes in conductivity after transition metal doping were also evaluated. Meanwhile, the different possibilities for the doping sites of the metal atoms were also considered. When the metal atoms substitute the two adjacent B atoms, the shape of the unit cell can maintain its initial shape (Figure S2a). However, when the metal atoms substitute the two nonadjacent B atoms, the shape of the unit cell deforms to rectangular and the pore size decreases, which is far away from the structure designed for a square shape (Figure S2b). Therefore, the doping mode for the adjacent B sites was ultimately chosen to form the B_2_M_2_ structure. The optimized structures of all transition-metal-doped systems are shown in Figure S4, and more structural information can be found in Table S1. Before calculating the material properties, formation energy is used to evaluate the stability of the structures. A negative formation energy indicates thermodynamic stability of the crystal structure. Therefore, the formation energies (*E*_f_) of these B_2_M_2_ candidate systems were calculated to assess their thermodynamic stability. Table S2 shows that the formation energies of these 14 systems are all negative, indicating that they are thermodynamically stable.

**Figure 2:**
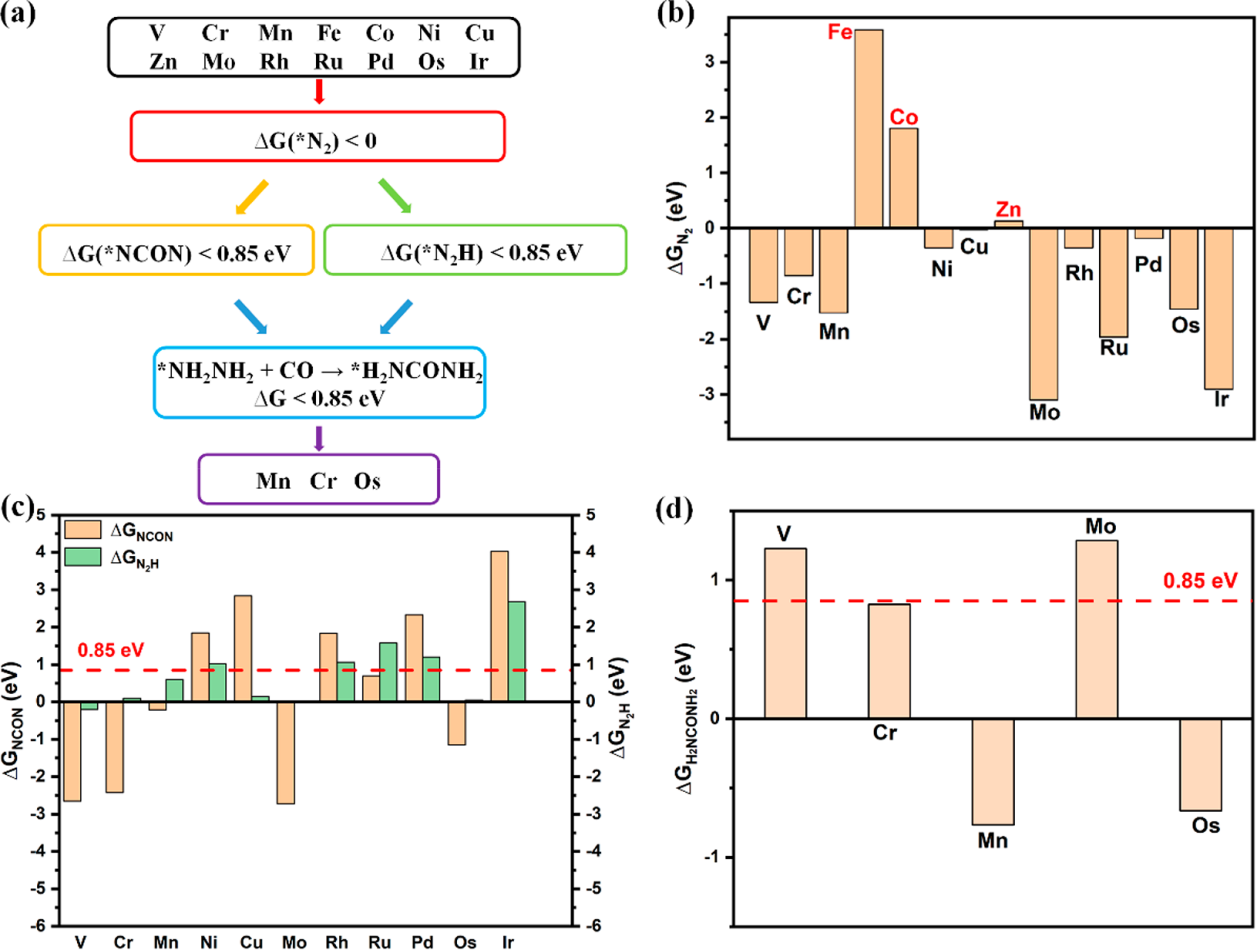
(a) Process flowchart for screening. (b) N_2_ adsorption energies of the 14 systems. (c) Free energy changes for the formation of *NCON and *N_2_H intermediates in 11 systems. (d) Free energy change for the coupling of H_2_*NN*H_2_ with CO to form H_2_*NCON*H_2_.

A possible synthetic pathway for B_2_M_2_ in experiments was designed, as shown in Figure S3. First, two −CN groups can be converted into a −C–N–C– to connect indoles.^46^ Then, by doping foreign boron atoms, the N atoms in indole can be replaced with B atoms. Finally, with doping transition metal atoms to substitute the adjacent two B atoms, the B_2_M_2_ structure is synthesized. Currently, doping substitution techniques have become quite mature and have been widely applied in various fields such as semiconductor materials, nanomaterials, ceramic materials, and metallic materials to improve material properties.^48^ Therefore, it is highly promising to achieve doping substitution at specified atomic positions. The synthesis of urea involves complex multistep reactions. In order to quickly screen appropriate electrocatalysts for urea, as shown in Figure 2a, the following strategies were formulated to screen 14 candidate systems while ensuring the system could exist stably: (1) the ability of the catalyst to adsorb N_2_ (Δ*G*(N_2_) < 0); (2) the free energy change of the first step of the reaction synthesizing urea after N_2_ adsorption via the NCON mechanism and CO mechanism; (3) the free energy change of the C–N coupling reaction of *CO and H_2_*NN*H_2_ intermediates. The free energy change values of (2) and (3) should not exceed 0.85 eV; otherwise, the catalytic performance of the catalyst in urea synthesis would be poor.

Effective adsorption of N_2_ is an important prerequisite for the efficient production of urea. N_2_ was adsorbed in a bridge-like configuration on bimetallic sites. The results of the N_2_ adsorption test are shown in Figure 2b. Apart from the B_2_Fe_2_, B_2_Co_2_, and B_2_Zn_2_ catalysts, where the adsorption energies of N_2_ were positive, the adsorption energies of N_2_ of the remaining systems were less than 0, indicating that the remaining 11 systems were capable of effectively adsorbing and activating N_2_. For the 11 systems remaining after the first screening, the free energy changes of the first reaction after N_2_ adsorption via the NCON and CO mechanism were tested, as shown in Figure 2c. If the energy required for the first reaction to occur is already higher than 0.85 eV, this already indicates poor performance in catalyzing urea synthesis under this mechanism. For the NCON mechanism, the first reaction after N_2_ adsorption is the coupling reaction of N_2_ and CO to form the *NCON* intermediate. By calculating the free energy change of forming the *NCON* intermediate, it was found that for B_2_Ni_2_, B_2_Cu_2_, B_2_Rh_2_, B_2_Pd_2_, and B_2_Ir_2_ catalysts, the free energy change of forming the *NCON* intermediate is greater than the standard of 0.85 eV (B_2_Ni_2_: 1.84 eV, B_2_Cu_2_: 2.84 eV, B_2_Rh_2_: 1.84 eV, B_2_Pd_2_: 2.32 eV, B_2_Ir_2_: 4.03 eV), while the free energy change of forming the *NCON* intermediate in the remaining six systems is negative. For the CO mechanism, the first reaction after N_2_ adsorption is the protonation of **N_2_ to form the *NN*H intermediate. The free energy changes of forming the *NN*H intermediate in B_2_V_2_ and B_2_Mo_2_ catalysts are −0.21 and −0.01 eV, respectively. The remaining catalysts B_2_Ni_2_, B_2_Pd_2_, B_2_Rh_2_, and B_2_Ir_2_ were excluded from the CO mechanism because the free energy change of forming the *NN*H intermediate is higher than 0.85 eV. Overall, after the second screening, the suitable candidate systems are B_2_V_2_, B_2_Cr_2_, B_2_Mn_2_, B_2_Mo_2_, and B_2_Os_2_.

Finally, the C–N coupling reaction in the CO pathway is also crucial. Therefore, the final screening was also tested based on the free energy change of CO inserting into the H_2_*NN*H_2_ intermediate to form urea being less than 0.85 eV, and the screening results are shown in Figure 2d. It can be observed that the free energy changes of CO coupling with H_2_*NN*H_2_ on B_2_V_2_ and B_2_Mo_2_ catalysts are higher than 0.85 eV, indicating that at least 0.85 eV of energy needs to be provided to synthesize urea and that they are not excellent catalysts for urea synthesis. The free energy changes of this step on B_2_Mn_2_ and B_2_Os_2_ catalysts are −0.76 and −0.66 eV, respectively. The Δ*G* value of this step on the B_2_Cr_2_ catalyst is 0.82 eV, which also meets the requirements. Therefore, B_2_Mn_2_, B_2_Cr_2_, and B_2_Os_2_ catalysts were finally found as suitable catalysts for urea synthesis. The electronic band structures for the B_2_Cr_2_, B_2_Mn_2_, and B_2_Os_2_ catalysts are shown in Figures S5, S6, and S7. The B_2_Cr_2_, B_2_Mn_2_, and B_2_Os_2_ catalysts exhibit metallic properties without a band gap near the Fermi level. This indicates that the conductivity of the catalysts is beneficial for electrocatalysis to produce urea.

**Figure 3:**
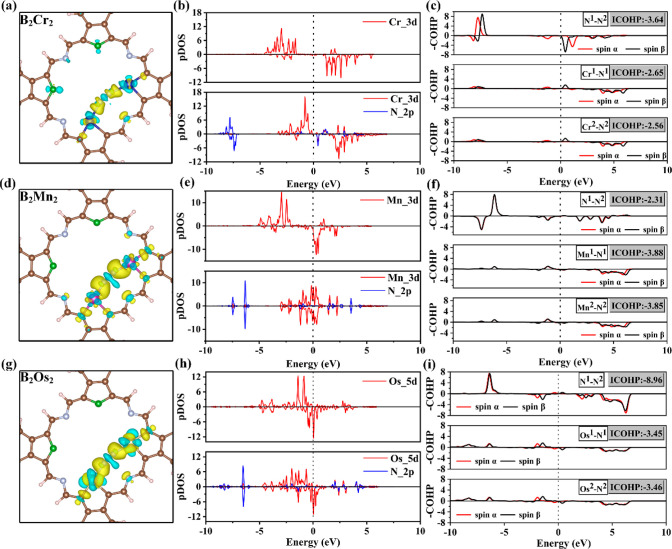
Charge difference density, pDOS, and COHP of absorbed N_2_ with a side-on pattern on bimetallic sites on (a–c) B_2_Cr_2_, (d–f) B_2_Mn_2_, and (g–i) B_2_Os_2_. In the pDOS diagram, “N” represents the nitrogen atom of N_2_. The contour level is set to 0.005 e/Å^3^.

The optimized structures of N_2_ adsorbed on bimetallic sites on B_2_Cr_2_, B_2_Mn_2_, and B_2_Os_2_ catalysts are shown in Figure S8. N_2_ and metal atoms are labeled as N^1^, N^2^, M^1^, and M^2^. To gain a deeper understanding of the mechanism of N_2_ activation on bimetallic sites, the electronic structure analysis of N_2_ adsorption was conducted, as show in Figure 3. The differential charge density of N_2_ adsorbed on the bimetallic sites was first calculated. The yellow part indicates an increase in charge density, while the blue part indicates a decrease in charge density. Both N atoms of N_2_ gain electrons (yellow), which proves that when N_2_ is adsorbed with a side-on pattern, the occupied d orbitals of the metal atoms on both sides provide electrons to the π* orbitals of N_2_. An increase in electrons can also be observed on the metal side, where the empty d orbitals of the metal can accept the lone pair electrons of N_2_. The changes in charge density on the two metal sites are similar, indicating that the two metal atoms in the same catalyst have similar adsorption capabilities for N_2_. Moreover, the yellow area near N_2_ on the B_2_Mn_2_ catalyst is the largest, so the adsorption effect of N_2_ on the B_2_Mn_2_ catalyst is stronger. The changes of charge density at Cr and N_2_ on the B_2_Cr_2_ catalyst are weaker than those on the B_2_Mn_2_ and B_2_Os_2_ catalysts, so the adsorption effect of N_2_ is the weakest. Then a pDOS and an integrated crystal orbital Hamilton population (ICOHP)^47^ analysis of Cr/Mn/Os-N_2_ and N_2_ adsorbed molecules was performed. The partial overlap of the d orbitals of metal with the 2p orbital of N_2_ indicates the bonding between the metal and N atom, and d orbitals of the metal accept the lone pair electrons of N_2_, resulting in a new peak of d orbitals at the Fermi level. The more negative the ICOHP value, the stronger the bond. The ICOHP value of free N_2_ is −22.97, as shown in Figure S9. It can be seen that the ICOHP values of the *NN* bond on B_2_Cr_2_ and B_2_Mn_2_ catalysts are −3.64 and −2.31, respectively, due to the activation of the N≡N bond by electrons from the 3d orbitals of Cr/Mn to the π* orbitals of N_2_. Although the 5d orbitals of Os also provide electrons to activate the N≡N bond, the N≡N bond is still strong (−8.96). The ICOHP values of bonds between the metal on both sides and N are very similar, which is consistent with what was observed in the differential charge density plot. Comparing the ICOHP values of Cr–N, Mn–N, and Os–N with the adsorption energy of N_2_ reveals that they are positively correlated, as shown in Figure S10.

The structure of N_2_ adsorbed between B and metal sites is shown in Figure S11. The electronic structure analysis of N_2_ adsorbed between B and metal sites was also performed (Figure S12). The differential charge density of N_2_ adsorbed between B and metal sites reveals that there is an accumulation of electrons at both N atoms on either side of N_2_. This proves that both the metal atom and the B atom provide electrons to the antibonding orbitals of N_2_ to weaken the NN bond. The charge density at Cr/Mn/Os decreases less, while the charge density at B decreases more. The electrons in the antibonding orbitals of N_2_ mainly come from the boron side. When N_2_ is adsorbed between B and the metal, the changes of charge density at both ends of N_2_ on the three catalysts are similar, so the adsorption energy of N_2_ is also similar. Similarly, partial density of states (pDOS) and integrated crystal orbital Hamilton population (ICOHP) analyses were also performed. The d orbitals of the metal, the 2p orbitals of B, and the 2p orbitals of N_2_ partially overlap. After N_2_ adsorption, the occupied d orbitals of Cr^2^, Mn^2^, and Os^2^ will provide electrons to the 2π* orbitals of N_2_ to form a new d–2π* orbital peak near the Fermi level. The unoccupied 2p orbitals of B interact with the π* orbitals of N_2_ to form new 2p−π* orbitals, causing the 2p orbital peak near the Fermi level to move toward lower energy levels. According to a comparison of ICOHP values for Cr–N/Mn–N/Os–N and B–N, there is a stronger bond between B^1^ atoms and N^1^, which also cause the adsorption strength of N_2_ to be greater than that on bimetallic sites. Similar to adsorption on bimetallic sites, Cr/Mn and B atoms work together to activate the N≡N bond, with ICOHP values of −3.93 and −1.73, respectively. Although Os and B can also activate the N≡N bond, it is still very strong. As shown in Figure S13, there is a positive correlation between ICOHP values for Cr-N/Mn-N/Os-N, B-N, and N_2_ adsorption energy.

In the end, the possibility of N_2_ being adsorbed between two B atoms was also considered. The structural optimization calculation by placing N_2_ between the two B atoms was performed, and it was found that due to the large distance between the two B atoms, N_2_ cannot form stable bonds with two B atoms simultaneously, as shown in Figure S14.

**Figure 4:**
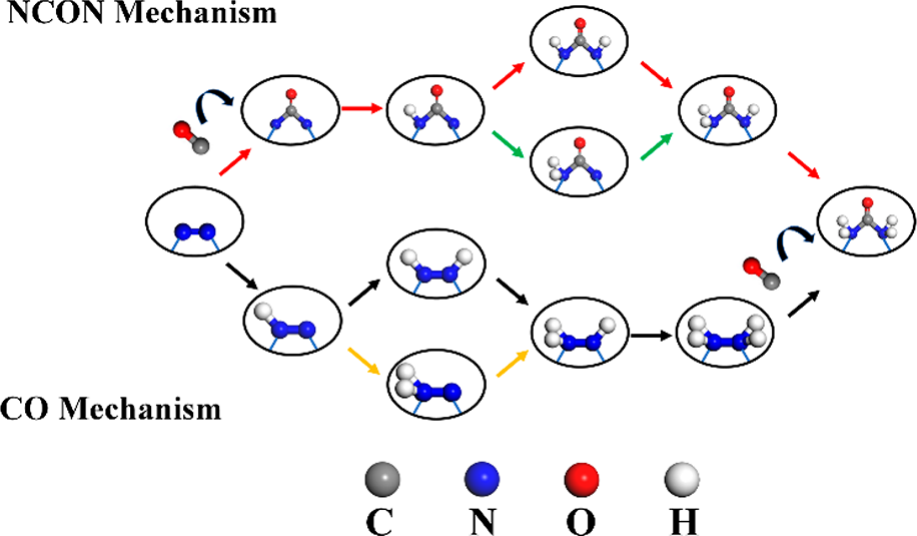
Schematic diagram for the NCON and CO mechanisms for urea synthesis within the pore.

Previously, the mechanism of urea synthesis was performed on the surface of the catalyst. In this study, a new mechanism where free CO is inserted for C–N coupling within the two-dimensional porous structure is proposed, which is named as the NCON and CO mechanisms. As shown in Figure 4, the NCON mechanism mainly involves the coupling of CO and N_2_ to form the *NCON* intermediate, and then the *NCON* species can be further reduced to urea through four proton-coupled electron transfer steps after the distal or alternative pathway in the pore. The CO mechanism mainly involves the further reduction of N_2_ to H_2_*NN*H_2_ through four proton-coupled electron transfer steps after the distal or alternating pathway, and then CO couples with H_2_*NN*H_2_ to form urea in the pore.

**Figure 5:**
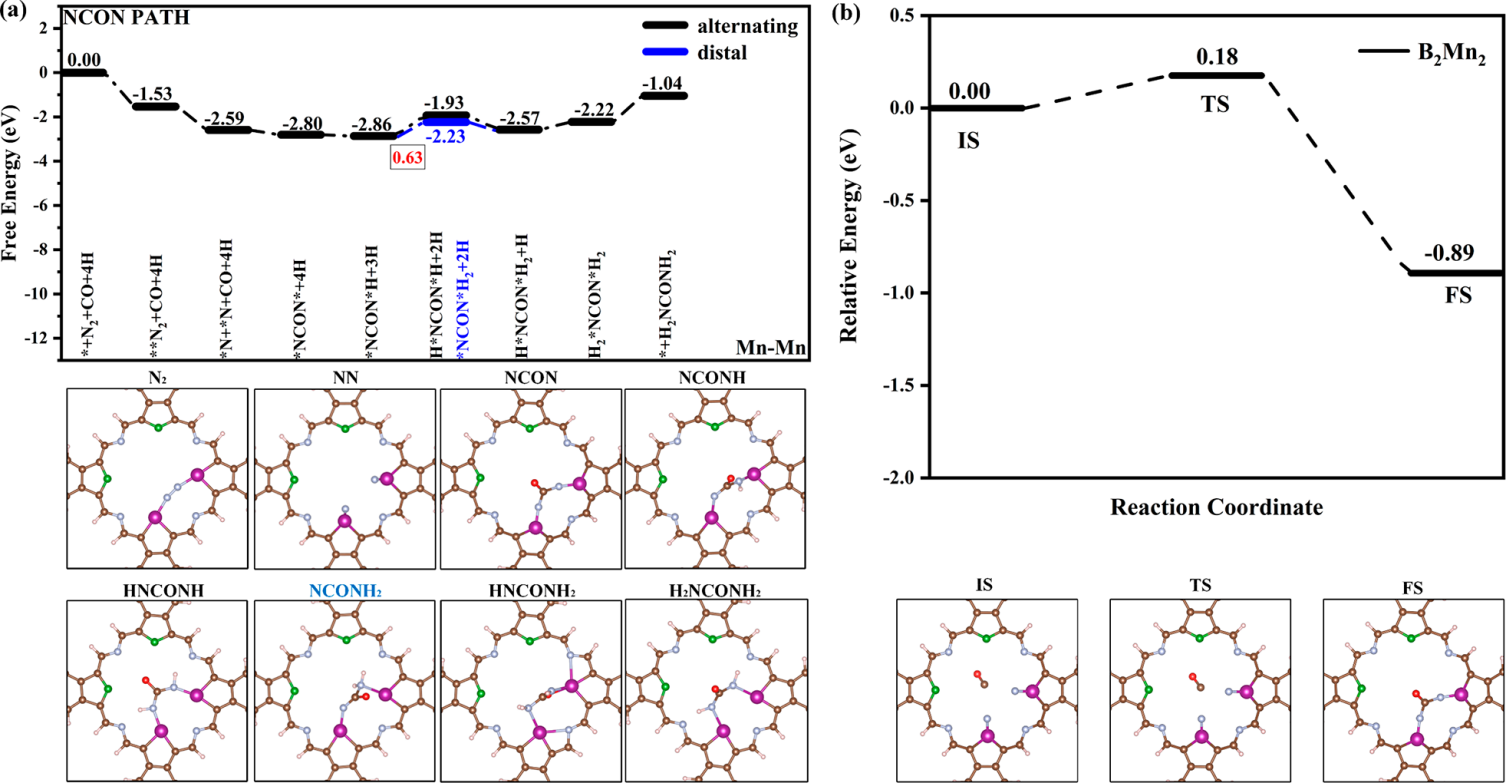
(a) Pathway diagram for urea synthesis via the NCON mechanism on Mn sites of the B_2_Mn_2_ system and intermediate configurations. (b) Representative configurations and corresponding energy barriers along the kinetic pathways of C–N coupling into *NCON* on B_2_Mn_2_.

The reaction paths were analyzed based on the bidentate metal–metal site and boron–metal site, respectively, and the mechanism was investigated based on both NCON and CO mechanisms. The performance of synthesizing urea through the NCON mechanism on bimetallic sites was first explored. The information about the free energy change of the basic steps of urea synthesis is shown in Table S3. The corresponding free energy change paths and the optimized configurations of the intermediates during the reaction process on B_2_Cr_2_ (Figure S15), B_2_Mn_2_ (Figure 5), and B_2_Os_2_ (Figure S16) catalysts are also presented. After N_2_ adsorption, on B_2_Mn_2_ and B_2_Cr_2_ catalysts, the N≡N bond can be broken first. The energy barriers required for the reaction to occur through CI-NEB were calculated, as shown in Figure S17. The energy barriers for the dissociation of the N≡N bond on B_2_Cr_2_ and B_2_Mn_2_ catalysts are 0.14 and 0.71 eV, indicating that the dissociation of the N≡N bond is thermodynamically feasible. *NCON* formed on B_2_Cr_2_, B_2_Mn_2_, and B_2_Os_2_ catalysts has free energy changes of −2.42, −0.21, and −1.15 eV, respectively. *NCON* is a key intermediate in urea synthesis, so the transition state energy barriers of C–N coupling reactions on three catalysts were explored. The transition state energy barriers for forming *NCON* on B_2_Cr_2_, B_2_Mn_2_, and B_2_Os_2_ catalysts are 1.76 eV (Figure S18a), 0.18 eV (Figure 5b), and 2.54 eV (Figure S18b), respectively. Relatively speaking, the coupling of CO with N_2_ is more challenging on the B_2_Cr_2_ catalyst, while it is relatively easier on the B_2_Mn_2_ and B_2_Os_2_ catalysts. Next, the hydrogenation step of *NCON* to *NCON*H requires the free energy change of 0.60 and 0.46 eV on B_2_Cr_2_ and B_2_Os_2_ catalysts, respectively, while on the B_2_Mn_2_ catalyst the free energy decreases by 0.06 eV. *NCON* → *NCON*H is the potential-limiting step for synthesizing urea through the NCON mechanism on Os sites, with a limiting potential of −0.46 V. There are two possible steps for subsequent hydrogenation of the *NCON*H intermediate: an alternating hydrogenation mechanism to form the H*NCON*H intermediate (black) and a distal hydrogenation mechanism to form the *NCON*H_2_ intermediate (blue). On B_2_Cr_2_ catalysts, forming the *NCON*H_2_ intermediate requires more free energy (1.49 eV) than forming the H*NCON*H intermediate (1.09 eV). Subsequently, the Δ*G* values of H*NCON*H → H*NCON*H_2_ and *NCON*H_2_ → H*NCON*H_2_ are 0.17 and −0.22 eV, respectively. This indicates that *NCON*H on B_2_Cr_2_ catalysts is more inclined to synthesize urea through the path of *NCON*H → H*NCON*H → H*NCON*H_2_. On B_2_Mn_2_ catalysts, it is easier to form the *NCON*H_2_ intermediate with a Δ*G* value of 0.63 eV. The formation process of the *NCON*H_2_ intermediate is the potential-limiting step for synthesizing urea through the NCON mechanism on B_2_Mn_2_ catalysts with a limiting potential of −0.63 V. The free energy changes for hydrogenating the H*NCON*H intermediate or hydrogenating the *NCON*H_2_ intermediate to form the H*NCON*H_2_ intermediate are −0.64 and −0.34 eV, respectively. On the B_2_Os_2_ catalyst, the formation of H*NCON*H and *NCON*H_2_ both release energy with Δ*G* values of −0.92 and −1.24 eV, respectively. However, the Δ*G* value of the subsequent hydrogenation of H*NCON*H to form H*NCON*H_2_ is 0.22 eV, which is less than that of the hydrogenation of *NCON*H_2_ to form H*NCON*H_2_ (0.53 eV). Overall, the process of *NCON*H → H*NCON*H → H*NCON*H_2_ is more likely to occur. The final step of hydrogenating H*NCON*H_2_ to form H_2_*NCON*H_2_ has a free energy change of 1.21, 0.35, and −0.16 eV on the three catalysts, respectively. The final step of hydrogenating H*NCON*H_2_ to form H_2_*NCON*H_2_ is also the potential-limiting step for the synthesis of urea via the NCON mechanism on the B_2_Cr_2_ catalyst, with a limiting potential of −1.21 V. In summary, the limiting potentials for urea synthesis via the NCON mechanism on bimetallic sites of B_2_Cr_2_, B_2_Mn_2_, and B_2_Os_2_ catalysts are −1.21 V (H*NCON*H_2_ → H_2_*NCON*H_2_), −0.63 V (*NCON*H → *NCON*H_2_), and −0.46 V (*NCON* → *NCON*H), respectively.

**Figure 6:**
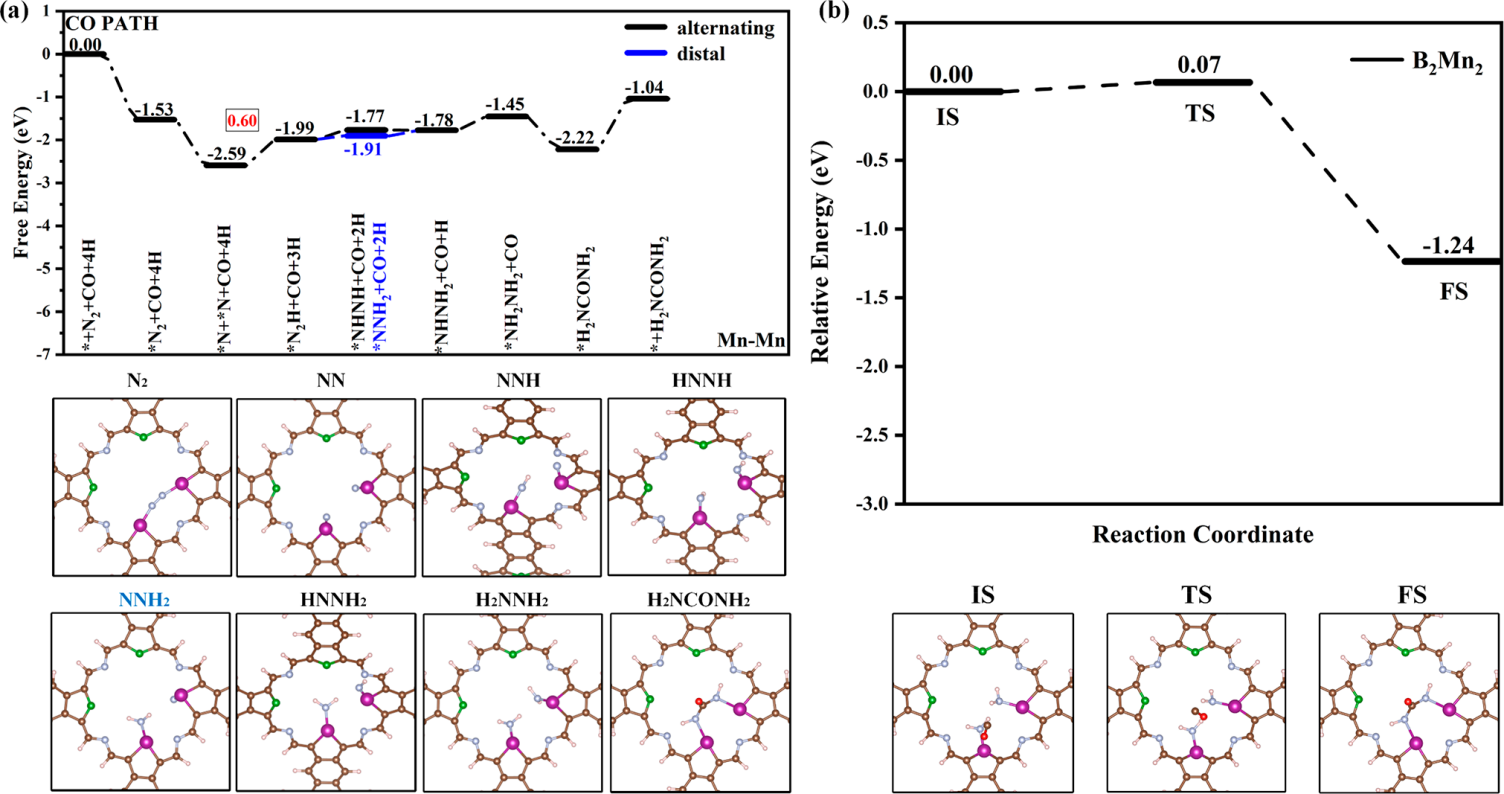
(a) Pathway diagram for urea synthesis via the CO mechanism on Mn sites of the B_2_Mn_2_ system and intermediate configurations. (b) Representative configurations and corresponding energy barriers along the kinetic pathways of the C–N coupling into H_2_*NCON*H_2_ on B_2_Mn_2_.

Next, the performance of coupling N_2_ and CO in synthesizing urea via the CO mechanism between bimetallic sites was calculated. The information on the free energy changes of the basic steps of urea synthesis is shown in Table S4. The corresponding reaction paths and the optimized configurations of the intermediates during the reaction process on B_2_Cr_2_ (Figure S19), B_2_Mn_2_ (Figure 6a), and B_2_Os_2_ (Figure S20) are shown in the Supporting Information. The free energy changes for the formation of *NN*H are 0.10 0.60, and 0.05 eV, suggesting that the formation of *NN*H is more challenging compared to *NCON* (−2.42, −0.21, and −1.15 eV). The formation process of *NN*H is the potential-limiting step for the synthesis of urea by the CO mechanism on the B_2_Mn_2_ catalyst, with a limiting potential of −0.60 V. The next step is to hydrogenate *NN*H into two paths: hydrogenating to the *NN*H_2_ intermediate or H*NN*H intermediate. On the B_2_Cr_2_, B_2_Mn_2_, and B_2_Os_2_ catalysts, the free energy required to form the H*NN*H intermediate is 0.28 0.22, and 0.42 eV, respectively. The energy required to form the *NN*H_2_ intermediate is lower with the free energy change of −0.25 and 0.08 eV respectively for B_2_Cr_2_ and B_2_Mn_2_ catalysts. For the B_2_Cr_2_ catalyst, hydrogenating *NN*H_2_ to form H*NN*H_2_ requires more free energy (0.56 eV) than that of hydrogenating H*NN*H to H*NN*H_2_ (0.03 eV). For the B_2_Os_2_ catalyst, the energy required to form the *NN*H_2_ intermediate is higher (0.79 eV). Therefore, for B_2_Cr_2_ and B_2_Os_2_ catalysts, it prefers to form urea through *NN*H → H*NN*H → H*NN*H_2_, while for the B_2_Mn_2_ catalyst, it has a tendency to form urea through *NN*H → *NN*H_2_ → H*NN*H_2_. Among them, the formation of the H*NN*H intermediate is a potential-determining step for synthesizing urea by the CO mechanism on the Os sites with a limiting potential of −0.42 V. The final hydrogenation step to form H_2_*NN*H_2_ has the Δ*G* values of −0.58, 0.32, and −1.05 eV for the three catalysts, respectively. Once H_2_*NN*H_2_ is formed, the free energy changes for CO insertion into the middle of H_2_*NN*H_2_ to synthesize urea are 0.83, −0.77, and −0.66 eV, respectively. The C–N coupling step is also the potential-limiting step for urea synthesis on the B_2_Cr_2_ catalyst, with a limiting potential of −0.83 V. To verify the feasibility of C–N coupling via the CO mechanism on the three catalysts, we conducted CI-NEB calculations. The transition state energy barriers for CO coupling with H_2_*NN*H_2_ on B_2_Cr_2_, B_2_Mn_2_, and B_2_Os_2_ catalysts are 0.05 eV (Figure S21a), 0.07 eV (Figure 6b), and 0.05 eV (Figure S21b), illustrating that the reaction is readily prone to occur. In summary, the limiting potentials for urea synthesis via the CO mechanism on bimetallic sites of B_2_Cr_2_, B_2_Mn_2_, and B_2_Os_2_ catalysts are −0.83, −0.60, and −0.42 V, respectively, so the CO mechanism is more likely to occur compared to the NCON mechanism (−1.21, −0.63, and −0.46 V) on the three catalysts.

**Figure 7:**
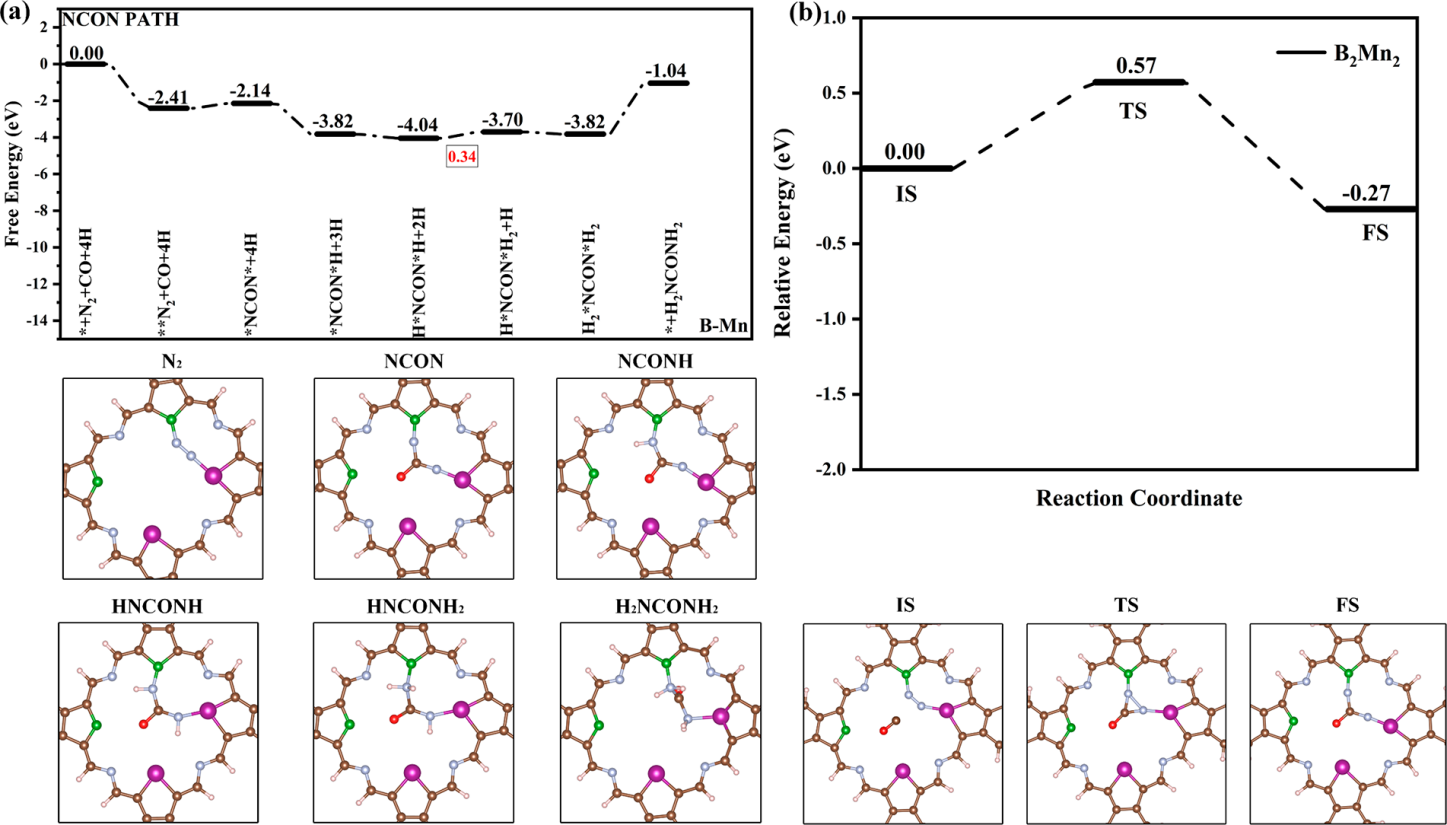
(a) One of the optimal pathways for urea synthesis via the NCON mechanism on B and Mn sites on the B_2_Mn_2_ catalyst. (b) Representative configurations and corresponding energy barriers along the kinetic pathways of C–N coupling into *NCON* on B_2_Mn_2_.

Besides bimetal sites, the mechanism of the bidentate site between boron and metal sites was also considered here, which is divided into NCON and CO mechanisms as well. The performances of the three catalysts in synthesizing urea through the NCON mechanism on the bidentate B and metal sites were explored first. The information about the free energy changes for urea synthesis is shown in Table S5, and the corresponding reaction paths of B_2_Cr_2_ (Figure S22a), B_2_Mn_2_ (Figure S23), and B_2_Os_2_ (Figure S24a) are shown in the Supporting Information. Once N_2_ is adsorbed, the free energy change of forming *NCON* on B_2_Cr_2_, B_2_Mn_2_, and B_2_Os_2_ catalysts are −1.76, −0.27, and 0.49 eV, respectively. Similarly, CI-NEB calculations were performed on the coupling of CO and N_2_ on metal–boron sites of the three catalysts, as shown in Figure S25 and Figure 7b. The transition state energy barriers for forming *NCON* on B_2_Cr_2_, B_2_Mn_2_, and B_2_Os_2_ catalysts are 0.10, 0.57, and 0.13 eV, respectively. In this case, the two adsorption sites for N_2_ are different, so unlike bimetallic sites, a different order of hydrogenation is considered. Therefore, there are two possibilities for the subsequent four-step hydrogenation of the *NCON* intermediate: hydrogenating the N bonded on the B side first or hydrogenating the N on the metal side first. Preferentially hydrogenating the N atom on the B side was first considered, and the reaction pathway is *NCON* → *NCON*H → H*NCON*H /*NCON*H_2_ → H*NCON*H_2_ → H_2_*NCON*H_2_. For B_2_Cr_2_, B_2_Mn_2_, and B_2_Os_2_ catalysts, the Δ*G* values for hydrogenating *NCON* to form the *NCON*H intermediate are −0.74, −1.67, and −1.56 eV, respectively. Next, hydrogenating *NCON*H to form H*NCON*H is energy-releasing, with Δ*G* values of −0.69, −0.22, and −0.49 eV, respectively. However, the Δ*G* values of forming the *NCON*H_2_ intermediate are 0.82, 0.44, and 0.28 eV, respectively. In terms of B_2_Cr_2_ and B_2_Mn_2_ catalysts, hydrogenating the H*NCON*H intermediate to form the H*NCON*H_2_ intermediate requires energy input, with values of 1.37 and 0.34 eV, respectively. While on B_2_Os_2_ catalysts, the energy change for forming the H*NCON*H_2_ intermediate is −0.09 eV. The final step of hydrogenation to form the H_2_*NCON*H_2_ intermediate on B_2_Cr_2_, B_2_Mn_2_, and B_2_Os_2_ catalysts has free energy changes of 0.85, −0.12, and 0.99 eV, respectively. Then preferentially adding H to the N atom of N_2_ on the metal side was also considered, and the reaction pathway on the metal side is *NCON* → H*NCON* → H*NCON*H/H_2_*NCON* → H_2_*NCON*H → H_2_*NCON*H_2_. The free energy changes for hydrogenating to form H*NCON* are 0.52, −0.26, and −1.89 eV, respectively. For all three catalysts, the Δ*G* values for forming the H*NCON*H intermediate or H_2_*NCON* intermediate are all negative. On B_2_Cr_2_ catalysts, hydrogenating H_2_*NCON* to form H_2_*NCON*H is easier, with a free energy change of 0.52 eV, and the other two catalysts are more inclined to hydrogenate the H*NCON*H intermediate to form the *H_2_*NCON*H intermediate. Finally, the free energy changes of hydrogenating the *H_2_*NCON*H intermediate to form the H_2_*NCON*H_2_ intermediate on B_2_Cr_2_, B_2_Mn_2_, and B_2_Os_2_ catalysts are 1.30, 0.61, and 1.17 eV, respectively. Moreover, we also found that there are two other possible pathways to synthesize urea: **N_2_ → *NCON* → *NCON*H → H*NCON*H → H_2_*NCON*H → H_2_*NCON*H_2_ and **N_2_ → *NCON* → *H*NCON* → H*NCON*H → H*NCON*H_2_ → H_2_*NCON*H_2_. For the first pathway, the limiting potentials for the synthesis of urea with B_2_Cr_2_, B_2_Mn_2_, and B_2_Os_2_ catalysts are 1.30, 0.61, and 1.17 eV, respectively. For the second pathway, the limiting potentials for urea synthesis with B_2_Cr_2_, B_2_Mn_2_, and B_2_Os_2_ catalysts are 1.37, 0.34, and 0.99 eV, respectively. In summary, when synthesizing urea through the NCON mechanism on B and metal sites, the optimal pathway for urea synthesis on the B_2_Cr_2_ catalyst is **N_2_ → *NCON* → *NCON*H → *NCON*H_2_ → H*NCON*H_2_ → H_2_*NCON*H_2_, with a limiting potential of −0.85 V (Figure S22b); one of the optimal reaction paths on the B_2_Mn_2_ catalyst is **N_2_ → *NCON* → *NCON*H → H*NCON*H → H*NCON*H_2_ → H_2_*NCON*H_2_, with a limiting potential of −0.34 V (Figure 7a). One of the optimal reaction pathways on the B_2_Os_2_ catalyst is **N_2_ → *NCON* → *H*NCON* → H*NCON*H → H*NCON*H_2_ → H_2_*NCON*H_2_, where the process of H*NCON*H_2_ → H_2_*NCON*H_2_ is a potential-limiting step with a limiting potential of −0.99 V (Figure S24b).

The performance of the three catalysts in synthesizing urea through the CO mechanism on B and the metal site was also explored. The information about the reaction path of urea synthesis is shown in Table S6, and the corresponding free energy change paths of B_2_Cr_2_ (Figure S26a), B_2_Mn_2_ (Figure S27a), and B_2_Os_2_ (Figure S28a) are shown in the Supporting Information. Similarly, we also first considered preferential hydrogenation of the N atom of N_2_ on the B side: *NN*H → H*NN*H/*NN*H_2_ → H*NN*H_2_ → H_2_*NN*H_2_ → H_2_*NCON*H_2_. For B_2_Cr_2_, B_2_Mn_2_, and B_2_Os_2_ catalysts, the Δ*G* values for hydrogenation to form *NN*H are −0.93, 0.45, and −0.33 eV, respectively. The next step is to hydrogenate *NN*H with two possible mechanisms: alternating hydrogenation to form H*NN*H or distal hydrogenation to form the *NN*H_2_ intermediate. For B_2_Cr_2_, B_2_Mn_2_, and B_2_Os_2_ catalysts, the energy required to form H*NN*H is lower than that for *NN*H_2_, which are 0.48, −0.29, and 0.62 eV, respectively. For B_2_Cr_2_ and B_2_Mn_2_ catalysts, the free energy changes for the formation of H*NN*H_2_ are 0.53 and 0.65 eV, respectively. In contrast, for the B_2_Os_2_ catalyst, it decreases to a value of −1.54 eV. The final proton transfer step to form H_2_*NN*H_2_ for the three catalysts is energy-releasing, with the free energy change of −2.82, −0.24, and −0.39 eV, respectively. Then CO is inserted into the H_2_*NN*H_2_ intermediate to form H_2_*NCON*H_2_ with a Δ*G* value of 1.79, −1.97, and 0.97 eV, respectively, so the formation of H_2_*NCON*H_2_ is less likely to occur for the B_2_Cr_2_ catalyst. We then considered preferentially adding a H atom to the N atom of N_2_ on the metal side: H*NN* → H*NN*H/H_2_*NN* → H_2_*NN*H → H_2_*NN*H_2_ → H_2_*NCON*H_2_. For the three catalysts, forming H*NN* is more challenging than forming *NN*H, with a free energy change of 0.04, 0.89, and 1.11 eV, respectively. Similarly, the second proton transfer step is also more facile to form H*NN*H, with a free energy change of −0.50, −0.74, and −0.82 eV for the B_2_Cr_2_, B_2_Mn_2_, and B_2_Os_2_ catalyst, respectively. Then hydrogenation of H*NN*H to form H_2_*NN*H is relatively feasible on the B_2_Mn_2_ catalyst with the free energy change of −0.20 eV, while for B_2_Cr_2_ and B_2_Os_2_ catalysts, it is slightly demanding, requiring a free energy change of 0.20 and 0.04 eV, respectively. Finally, hydrogenation of the H_2_*NN*H intermediate to form the H_2_*NN*H_2_ intermediate is feasible on B_2_Cr_2_ and B_2_Os_2_ catalysts with Δ*G* values of −2.49 and −1.96 eV, respectively; on the other hand, it is relatively challenging for the B_2_Mn_2_ catalyst, with a free energy change of 0.61 eV. Similar to the NCON pathway, the possibilities of H*NN* → H*NN*H → H*NN*H_2_ → H_2_*NN*H_2_ → H_2_*NCON*H_2_ and *NN*H → H*NN*H → H_2_*NN*H → H_2_*NN*H_2_ → H_2_*NCON*H_2_ have also been considered. For the first pathway, the limiting potentials for urea synthesis on B_2_Cr_2_, B_2_Mn_2_, and B_2_Os_2_ are 1.79, 0.61, and 0.97 eV, respectively. Regarding the second pathway, the limiting potentials for urea synthesis on these three catalysts are 1.79, 0.89, and 1.11 eV. Generally, when synthesizing urea via the CO mechanism on B and metal sites, the best reaction path on the B_2_Cr_2_ catalyst is **N_2_ → H*NN* → H*NN*H → H*NN*H_2_ → H_2_*NN*H_2_ → H_2_*NCON*H_2_, with a limiting potential of −1.79 V (Figure S26b); one of the best reaction paths on the B_2_Mn_2_ catalyst is **N_2_ → *NN*H → H*NN*H → H_2_*NN*H → H_2_*NN*H_2_ → H_2_*NCON*H_2_, with a limiting potential of −0.61 V (Figure S27b). One of the best reaction paths on the B_2_Os_2_ catalyst is **N_2_ → *NN*H → H*NN*H → H*NN*H_2_ → H_2_*NN*H_2_ → H_2_*NCON*H_2_, with a limiting potential of −0.97 V (Figure S28b). Finally, CI-NEB calculations were also performed for the coupling of CO to H_2_*NN*H_2_ on boron–metal sites, as shown in Figure S29. The results showed that the transition state energy barriers for the C–N coupling reaction on B_2_Cr_2_, B_2_Mn_2_, and B_2_Os_2_ catalysts are 1.19, 1.37, and 1.20 eV, respectively.

Nitrogen reduction reaction (NRR) is a key competitive reaction for urea synthesis. To ensure high selectivity for urea synthesis, the limiting potential for urea synthesis should be greater than the limiting potential for competitive NRR. Therefore, the competitive NRR reaction on the three catalysts was evaluated, as shown in Figures S30, S31, and S32. On B_2_Cr_2_, B_2_Mn_2_, and B_2_Os_2_ catalysts, the limiting potentials for the best NRR reaction paths are −0.96, −0.60, and −0.62 V, respectively, all lower than the optimal limiting potentials for urea synthesis on the three catalysts: −0.83, −0.34, and −0.42 V, indicating that NRR can be greatly suppressed on these three catalysts.

Then, the selectivity of urea synthesis in comparison to that of the production of C1 products through the CO reduction reaction (CORR) was also evaluated. The catalytic performance of CORR in forming CH_4_ on the three catalysts on the B site or metal site was separately assessed, which are shown in Figure S33, Figure S34, and Figure S35. The results showed that the optimal limiting potentials for CH_4_ production on the B_2_Cr_2_, B_2_Mn_2_, and B_2_Os_2_ catalysts are −1.49, −1.57, and −1.30 V, all lower than the optimal limiting potential for urea synthesis. Therefore, CORR is also effectively inhibited on the three catalysts.

## Conclusion

To recapitulate, we designed a new 2D porous carbon nitride material embedded with transition metals and boron. We developed a strategy for selecting an appropriate urea catalyst. Eventually, B_2_Cr_2_, B_2_Mn_2_, and B_2_Os_2_ were identified as suitable urea catalysts. The differential charge density and pDOS results show electron transfer between N_2_ and the metal or B on both sides. COHP analysis revealed that the bonding strength between N_2_ and B was greater than that between N_2_ and metal, and the N≡N bond of N_2_ was activated to some extent. We explored the NCON and CO mechanisms for the coupling of CO and N_2_ to synthesize urea on bidentate metal–metal sites and bidentate B–metal sites within a two-dimensional pore. A new mechanism where free CO is inserted for C–N coupling within the two-dimensional porous structure is proposed. The limiting potentials for the optimal path of urea synthesis on the B_2_Cr_2_, B_2_Mn_2_, and B_2_Os_2_ catalysts are −0.83, −0.34, and −0.42 V, respectively, while the transition state energy barriers for the corresponding C–N coupling reactions are 0.05, 0.57, and 0.05 eV. Furthermore, this study provides new structures for designing efficient electrocatalysts and new mechanisms within a two-dimensional porous material for urea synthesis.

## Supporting Information Available

Electronic properties, phonon spectrum, structural information, experimental synthesis path, pore size, formation energy, adsorption pattern, charge difference density, crystal orbital Hamilton population analyses, reaction path and intermediate, energy barrier diagrams, comparison with other catalysts, competitive reaction (PDF)

## References

[ref1] Wei X.; Wen X.; Liu Y.; Chen C.; Xie C.; Wang D.; Qiu M.; He N.; Zhou P.; Chen W.; Cheng J.; Lin H.; Jia J.; Fu X. Z.; Wang S. Oxygen Vacancy-Mediated Selective C-N Coupling toward Electrocatalytic Urea Synthesis. J. Am. Chem. Soc. 2022, 144 (26), 11530–11535. 10.1021/jacs.2c03452. 35748598

[ref2] Lim J.; Fernández C. A.; Lee S. W.; Hatzell M. C. Ammonia and Nitric Acid Demands for Fertilizer Use in 2050. ACS Energy Lett. 2021, 6 (10), 3676–3685. 10.1021/acsenergylett.1c01614.

[ref3] Yao Y.; Zhu S.; Wang H.; Li H.; Shao M. A Spectroscopic Study on the Nitrogen Electrochemical Reduction Reaction on Gold and Platinum Surfaces. J. Am. Chem. Soc. 2018, 140 (4), 1496–1501. 10.1021/jacs.7b12101. 29320173

[ref4] Kitano M.; Kanbara S.; Inoue Y.; Kuganathan N.; Sushko P. V.; Yokoyama T.; Hara M.; Hosono H. Electride support boosts nitrogen dissociation over ruthenium catalyst and shifts the bottleneck in ammonia synthesis. Nat. Commun. 2015, 6, 6731 10.1038/ncomms7731. 25816758 PMC4389256

[ref5] Suryanto B. H. R.; Du H.-L.; Wang D.; Chen J.; Simonov A. N.; MacFarlane D. R. Challenges and prospects in the catalysis of electroreduction of nitrogen to ammonia. Nat. Catal. 2019, 2 (4), 290–296. 10.1038/s41929-019-0252-4.

[ref6] vander Ham C. J.; Koper M. T.; Hetterscheid D. G. Challenges in reduction of dinitrogen by proton and electron transfer. Chem. Soc. Rev. 2014, 43 (15), 5183–91. 10.1039/C4CS00085D. 24802308

[ref7] Guo C.; Zhou W.; Lan X.; Wang Y.; Li T.; Han S.; Yu Y.; Zhang B. Electrochemical Upgrading of Formic Acid to Formamide via Coupling Nitrite Co-Reduction. J. Am. Chem. Soc. 2022, 144 (35), 16006–16011. 10.1021/jacs.2c05660. 35905476

[ref8] Chen C.; Zhu X.; Wen X.; Zhou Y.; Zhou L.; Li H.; Tao L.; Li Q.; Du S.; Liu T.; Yan D.; Xie C.; Zou Y.; Wang Y.; Chen R.; Huo J.; Li Y.; Cheng J.; Su H.; Zhao X.; Cheng W.; Liu Q.; Lin H.; Luo J.; Chen J.; Dong M.; Cheng K.; Li C.; Wang S. Coupling N_2_ and CO_2_ in H_2_O to synthesize urea under ambient conditions. Nat. Chem. 2020, 12 (8), 717–724. 10.1038/s41557-020-0481-9. 32541948

[ref9] Lv C.; Zhong L.; Liu H.; Fang Z.; Yan C.; Chen M.; Kong Y.; Lee C.; Liu D.; Li S.; Liu J.; Li S.; Chen G.; Yan Q.; Yu G. Selective electrocatalytic synthesis of urea with nitrate and carbon dioxide. Nat. Sustainability 2021, 4 (10), 868–876. 10.1038/s41893-021-00741-3.

[ref10] Yuan M.; Chen J.; Bai Y.; Liu Z.; Zhang J.; Zhao T.; Wang Q.; Li S.; He H.; Zhang G. Unveiling Electrochemical Urea Synthesis by Co-Activation of CO_2_ and N_2_ with Mott-Schottky Heterostructure Catalysts. Angew. Chem., Int. Ed. Engl. 2021, 60 (19), 10910–10918. 10.1002/anie.202101275. 33634560

[ref11] Mukherjee J.; Paul S.; Adalder A.; Kapse S.; Thapa R.; Mandal S.; Ghorai B.; Sarkar S.; Ghorai U. K. Understanding the Site-Selective Electrocatalytic Co-Reduction Mechanism for Green Urea Synthesis Using Copper Phthalocyanine Nanotubes. Adv. Funct. Mater. 2022, 32 (31), 2200882 10.1002/adfm.202200882.

[ref12] Yuan M.; Chen J.; Xu Y.; Liu R.; Zhao T.; Zhang J.; Ren Z.; Liu Z.; Streb C.; He H.; Yang C.; Zhang S.; Zhang G. Highly selective electroreduction of N_2_ and CO_2_ to urea over artificial frustrated Lewis pairs. Energy Environ. Sci. 2021, 14 (12), 6605–6615. 10.1039/D1EE02485J.

[ref13] Xue Z. H.; Zhang S. N.; Lin Y. X.; Su H.; Zhai G. Y.; Han J. T.; Yu Q. Y.; Li X. H.; Antonietti M.; Chen J. S. Electrochemical Reduction of N_2_ into NH_3_ by Donor-Acceptor Couples of Ni and Au Nanoparticles with a 67.8% Faradaic Efficiency. J. Am. Chem. Soc. 2019, 141 (38), 14976–14980. 10.1021/jacs.9b07963. 31523954

[ref14] Legare M.-A.; Belanger-Chabot G.; Dewhurst R. D.; Welz E.; Krummenacher I.; Engels B.; Braunschweig H. Nitrogen fixation and reduction at boron. Science 2018, 359 (6378), 896–899. 10.1126/science.aaq1684. 29472479

[ref15] Zou L.; Wei Y. S.; Hou C. C.; Li C.; Xu Q. Single-Atom Catalysts Derived from Metal-Organic Frameworks for Electrochemical Applications. Small 2021, 17 (16), e2004809 10.1002/smll.202004809. 33538109

[ref16] Zang W.; Yang T.; Zou H.; Xi S.; Zhang H.; Liu X.; Kou Z.; Du Y.; Feng Y. P.; Shen L.; Duan L.; Wang J.; Pennycook S. J. Copper Single Atoms Anchored in Porous Nitrogen-Doped Carbon as Efficient pH-Universal Catalysts for the Nitrogen Reduction Reaction. ACS Catal. 2019, 9 (11), 10166–10173. 10.1021/acscatal.9b02944.

[ref17] Gu Y.; Xi B.; Tian W.; Zhang H.; Fu Q.; Xiong S. Boosting Selective Nitrogen Reduction via Geometric Coordination Engineering on Single-Tungsten-Atom Catalysts. Adv. Mater. 2021, 33 (25), e2100429 10.1002/adma.202100429. 33998069

[ref18] Jiao L.; Zhu J.; Zhang Y.; Yang W.; Zhou S.; Li A.; Xie C.; Zheng X.; Zhou W.; Yu S. H.; Jiang H. L. Non-Bonding Interaction of Neighboring Fe and Ni Single-Atom Pairs on MOF-Derived N-Doped Carbon for Enhanced CO_2_ Electroreduction. J. Am. Chem. Soc. 2021, 143 (46), 19417–19424. 10.1021/jacs.1c08050. 34779627

[ref19] Zhang X.; Zhu X.; Bo S.; Chen C.; Qiu M.; Wei X.; He N.; Xie C.; Chen W.; Zheng J.; Chen P.; Jiang S. P.; Li Y.; Liu Q.; Wang S. Identifying and tailoring C-N coupling site for efficient urea synthesis over diatomic Fe-Ni catalyst. Nat. Commun. 2022, 13 (1), 5337 10.1038/s41467-022-33066-6. 36088335 PMC9464195

[ref20] Jiang M.; Zhu M.; Wang M.; He Y.; Luo X.; Wu C.; Zhang L.; Jin Z. Review on Electrocatalytic Coreduction of Carbon Dioxide and Nitrogenous Species for Urea Synthesis. ACS Nano 2023, 17 (4), 3209–3224. 10.1021/acsnano.2c11046. 36786415

[ref21] Zhao Y.; Ding Y.; Li W.; Liu C.; Li Y.; Zhao Z.; Shan Y.; Li F.; Sun L.; Li F. Efficient urea electrosynthesis from carbon dioxide and nitrate via alternating Cu-W bimetallic C-N coupling sites. Nat. Commun. 2023, 14 (1), 4491 10.1038/s41467-023-40273-2. 37495582 PMC10372083

[ref22] Lv C.; Lee C.; Zhong L.; Liu H.; Liu J.; Yang L.; Yan C.; Yu W.; Hng H. H.; Qi Z.; Song L.; Li S.; Loh K. P.; Yan Q.; Yu G. A Defect Engineered Electrocatalyst that Promotes High-Efficiency Urea Synthesis under Ambient Conditions. ACS Nano 2022, 16 (5), 8213–8222. 10.1021/acsnano.2c01956. 35362943

[ref23] Tao Z.; Rooney C. L.; Liang Y.; Wang H. Accessing Organonitrogen Compounds via C-N Coupling in Electrocatalytic CO_2_ Reduction. J. Am. Chem. Soc. 2021, 143 (47), 19630–19642. 10.1021/jacs.1c10714. 34787404

[ref24] Zhang X.; Zhu X.; Bo S.; Chen C.; Cheng K.; Zheng J.; Li S.; Tu X.; Chen W.; Xie C.; Wei X.; Wang D.; Liu Y.; Chen P.; Jiang S. P.; Li Y.; Liu Q.; Li C.; Wang S. Electrocatalytic Urea Synthesis with 63.5% Faradaic Efficiency and 100% N-Selectivity via One-step C-N coupling. Angew. Chem., Int. Ed. Engl. 2023, 62 (33), e202305447 10.1002/anie.202305447. 37337852

[ref25] Fu J.; Yang Y.; Hu J.-S. Dual-Sites Tandem Catalysts for C-N Bond Formation via Electrocatalytic Coupling of CO_2_ and Nitrogenous Small Molecules. ACS Mater. Lett. 2021, 3 (10), 1468–1476. 10.1021/acsmaterialslett.1c00375.

[ref26] Li X.; Liu L.; Ren X.; Gao J.; Huang Y.; Liu B. Microenvironment modulation of single-atom catalysts and their roles in electrochemical energy conversion. Sci. Adv. 2020, 6 (39), abb6833 10.1126/sciadv.abb6833. PMC7531890 32967833

[ref27] Chen S.; Yuan H.; Morozov S. I.; Ge L.; Li L.; Xu L.; Goddard W. A. 3rd Design of a Graphene Nitrene Two-Dimensional Catalyst Heterostructure Providing a Well-Defined Site Accommodating One to Three Metals, with Application to CO_2_ Reduction Electrocatalysis for the Two-Metal Case. J. Phys. Chem. Lett. 2020, 11 (7), 2541–2549. 10.1021/acs.jpclett.0c00642. 32163707

[ref28] Gao Y.; Zhang D.; Yuan H.; Min Y.; Chen S.; Xu L. Long distance bimetallic site in crystal with relay metal-N-N-metal mechanism and new descriptors for electrocatalytic nitrogen reduction reaction. Appl. Catal. A: Gen. 2023, 652, 119030 10.1016/j.apcata.2023.119030.

[ref29] Hering-Junghans C. Metal-Free Nitrogen Fixation at Boron. Angew. Chem., Int. Ed. Engl. 2018, 57 (23), 6738–6740. 10.1002/anie.201802675. 29718573

[ref30] Yu X.; Han P.; Wei Z.; Huang L.; Gu Z.; Peng S.; Ma J.; Zheng G. Boron-Doped Graphene for Electrocatalytic N_2_ Reduction. Joule 2018, 2 (8), 1610–1622. 10.1016/j.joule.2018.06.007.

[ref31] Gu C.; Hosono N.; Zheng J. J.; Sato Y.; Kusaka S.; Sakaki S.; Kitagawa S. Design and control of gas diffusion process in a nanoporous soft crystal. Science 2019, 363 (6425), 387 10.1126/science.aar6833. 30679369

[ref32] Wang S.; Xie Z.; Zhu D.; Fu S.; Wu Y.; Yu H.; Lu C.; Zhou P.; Bonn M.; Wang H. I.; Liao Q.; Xu H.; Chen X.; Gu C. Efficient photocatalytic production of hydrogen peroxide using dispersible and photoactive porous polymers. Nat. Commun. 2023, 14 (1), 6891 10.1038/s41467-023-42720-6. 37898686 PMC10613291

[ref33] Su Y.; Otake K. I.; Zheng J. J.; Horike S.; Kitagawa S.; Gu C. Separating water isotopologues using diffusion-regulatory porous materials. Nature 2022, 611 (7935), 289–294. 10.1038/s41586-022-05310-y. 36352136

[ref34] Su Y.; Li B.; Xu H.; Lu C.; Wang S.; Chen B.; Wang Z.; Wang W.; Otake K. I.; Kitagawa S.; Huang L.; Gu C. Multi-Component Synthesis of a Buta-1,3-diene-Linked Covalent Organic Framework. J. Am. Chem. Soc. 2022, 144 (40), 18218–18222. 10.1021/jacs.2c05701. 36069433

[ref35] Chen H.; Liang X.; Liu Y.; Ai X.; Asefa T.; Zou X. Active Site Engineering in Porous Electrocatalysts. Adv. Mater. 2020, 32 (44), e2002435 10.1002/adma.202002435. 32666550

[ref36] Kessler F. K.; Zheng Y.; Schwarz D.; Merschjann C.; Schnick W.; Wang X.; Bojdys M. J. Functional carbon nitride materials-design strategies for electrochemical devices. Nat. Rev. Mater. 2017, 2 (6), 17030 10.1038/natrevmats.2017.30.

[ref37] Eley D. D.; Rideal E. K. Parahydrogen conversion on tungsten. Nature 1940, 146, 401–402. 10.1038/146401d0.

[ref38] Xie L. S.; Skorupskii G.; Dincǎ M. Electrically Conductive Metal-Organic Frameworks. Chem. Rev. 2020, 120 (16), 8536–8580. 10.1021/acs.chemrev.9b00766. 32275412 PMC7453401

[ref39] Hafner J. Ab-initio simulations of materials using VASP: Density-functional theory and beyond. J. Comput. Chem. 2008, 29 (13), 2044–2078. 10.1002/jcc.21057. 18623101

[ref40] Kresse G.; Joubert D. From ultrasoft pseudopotentials to the projector augmented-wave method. Phys. Rev. B 1999, 59 (3), 1758–1775. 10.1103/PhysRevB.59.1758.

[ref41] Perdew J. P.; Burke K.; Ernzerhof M. Generalized gradient approximation made simple. Phys. Rev. Lett. 1996, 77 (18), 3865–3868. 10.1103/PhysRevLett.77.3865. 10062328

[ref42] Grimme S.; Antony J.; Ehrlich S.; Krieg H. A consistent and accurate ab initio parametrization of density functional dispersion correction (DFT-D) for the 94 elements H-Pu. J. Chem. Phys. 2010, 132 (15), 154104 10.1063/1.3382344. 20423165

[ref43] Mathew K.; Sundararaman R.; Letchworth-Weaver K.; Arias T. A.; Hennig R. G. Implicit solvation model for density-functional study of nanocrystal surfaces and reaction pathways. J. Chem. Phys. 2014, 140 (8), 084106 10.1063/1.4865107. 24588147

[ref44] Togo A.; Tanaka I. First principles phonon calculations in materials science. Scr. Mater. 2015, 108, 1–5. 10.1016/j.scriptamat.2015.07.021.

[ref45] Henkelman G.; Uberuaga B. P.; Jónsson H. A climbing image nudged elastic band method for finding saddle points and minimum energy paths. J. Chem. Phys. 2000, 113 (22), 9901–9904. 10.1063/1.1329672.

[ref46] Xie L. Y.; Boyle R. W.; Dolphin D. Porphocyanines: Expanded aromatic tetrapyrrolic macrocycles. J. Am. Chem. Soc. 1996, 118 (20), 4853–4859. 10.1021/ja953721k.

[ref47] Dronskowski R.; Blochl P. E. Crystal Orbital Hamilton Populations(COHP). Energy Resolved Visualization of Chemical Bonding in Solids Basedon Density-Functional Calculations. J. Phys. Chem. 1993, 97 (33), 8617–8624. 10.1021/j100135a014.

[ref48] Agnoli S.; Favaro M. Doping graphene with boron: a review of synthesis methods, physicochemical characterization, and emerging applications. J. Mater. Chem. A 2016, 4 (14), 5002–5025. 10.1039/C5TA10599D.

