# Supplementary material for: Design of Boron and Transition Metal Embedded Two-Dimensional Porous Carbon Nitride for Electrocatalytic Synthesis of Urea
Source: J Am Chem Soc. 2023 Dec 26;146(1):1042–52. doi: 10.1021/jacs.3c12017 (PMC10785813; doi:10.1021/jacs.3c12017)
Supplement: Supplementary file 1 — ja3c12017_si_001.pdf [file ja3c12017_si_001.pdf]

Design of Boron and Transition Metal Embedded Two-Dimensional Porous Carbon Nitride for Electrocatalytic Synthesis of Urea

Xin Cao[#1], Dewei Zhang[#1], Yongqi Gao[#1], Oleg V. Prezhdo[*2], Lai Xu[*1]

1 Institute of Functional Nano & Soft Materials (FUNSOM), Jiangsu Key Laboratory of Advanced Negative Carbon Technologies, Jiangsu Key Laboratory for Carbon-Based Functional Materials & Devices, Joint International Research Laboratory of Carbon-Based Functional Materials and Devices, Soochow University, Suzhou, 215123, Jiangsu, PR China

2 Department of Chemistry, University of Southern California, Los Angeles, California 90089, United States

Email: prezhdo@usc.edu; xulai15@suda.edu.cn

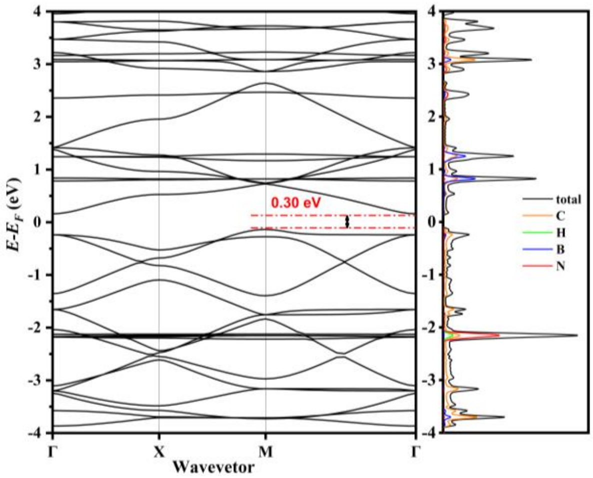

Figure S1.The elemental projected band and the DOS of N_4B_4 monolayers. The Fermi level is assigned at zero.

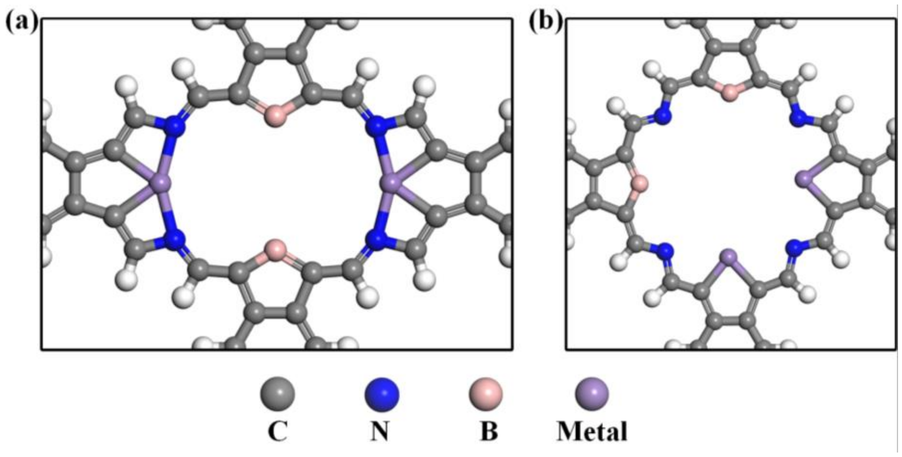

Figure S2. The relative position of transition metals and B atoms: (a) Optimized structures of two B atoms substituted with metals at the para position. (b) Optimized structures of two B atoms substituted with metals at the adjacent positions.

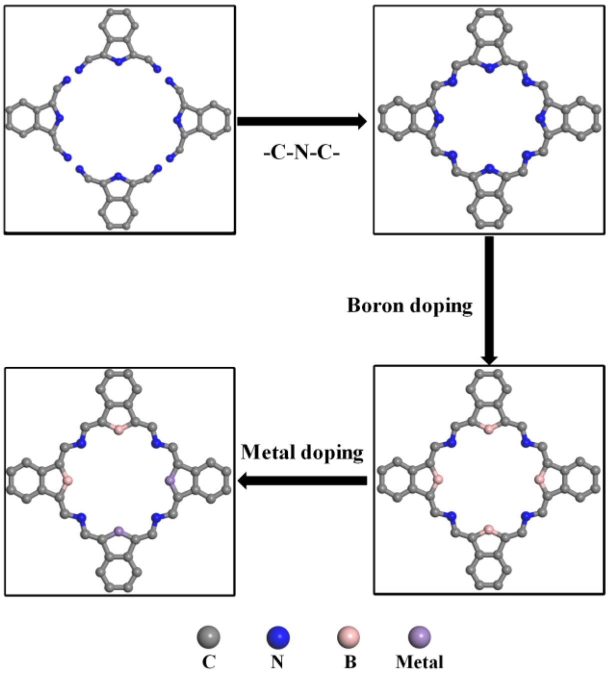

Figure S3. Possible pathways for the experimental synthesis of B_2M_2 structures.

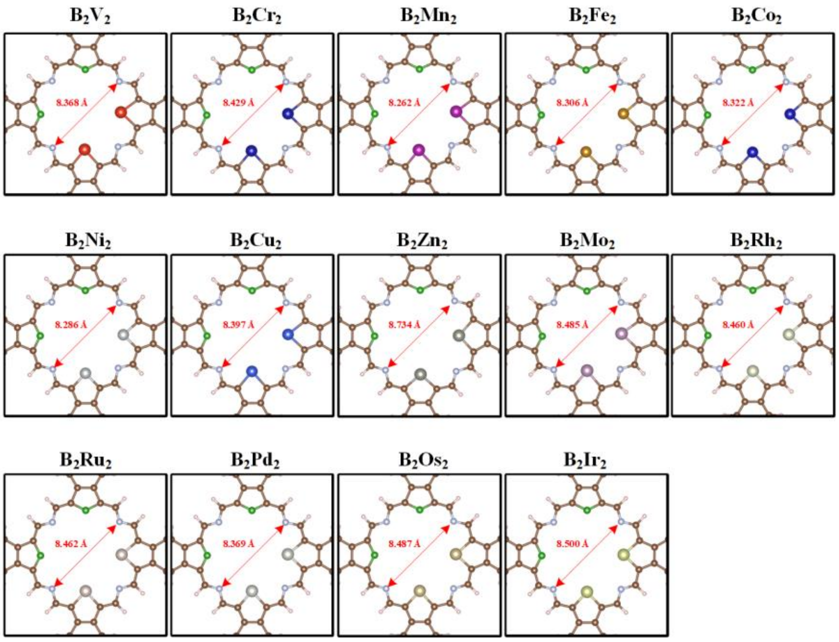

Figure S4. Top view of optimized structures of fourteen candidate catalysts.

Table S1. The d_{M-M} represents the distance between two adjacent metal atoms around the pore. The d_{B-M} represents the distance between two relatively close boron and metal atoms around the pore. The d_{B-B} represents the distance between two adjacent boron atoms around the pore.

System	d_{M-M}/Å	d_{B-M}/Å	d_{B-B}/Å
B_2V_2	4.622	4.943	5.627
B_2Cr_2	4.626	5.037	5.676
B_2Mn_2	4.764	4.984	5.612
B_2Fe_2	4.746	5.093	5.633
B_2Co_2	4.741	5.145	5.645
B_2Ni_2	4.759	5.142	5.644
B_2Cu_2	4.591	5.036	5.685
B_2Zn_2	4.872	5.322	5.772
B_2Mo_2	4.500	4.880	5.652
B_2Rh_2	4.578	5.084	5.682
B_2Ru_2	4.551	5.072	5.689
B_2Pd_2	4.576	5.057	5.664
B_2Os_2	4.586	5.091	5.690
B_2Ir_2	4.622	5.102	5.683

Table S2. Formation energies of 14 systems. (T=0 K)

System	E_f (eV)	System	E_f (eV)
B_2V_2	-4.91	B_2Zn_2	-4.96
B_2Cr_2	-4.94	B_2Mo_2	-4.87
B_2Mn_2	-4.95	B_2Ru_2	-4.89
B_2Fe_2	-4.93	B_2Rh_2	-4.93
B_2Co_2	-4.93	B_2Pd_2	-4.97
B_2Ni_2	-4.95	B_2Os_2	-4.84
B_2Cu_2	-5.13	B_2Ir_2	-4.91

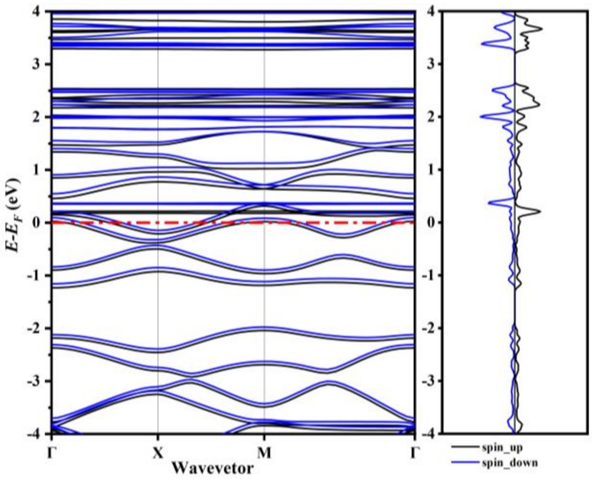

Figure S5. The band structure and the total density of states of B_2Cr_2. The Fermi level is assigned at zero.

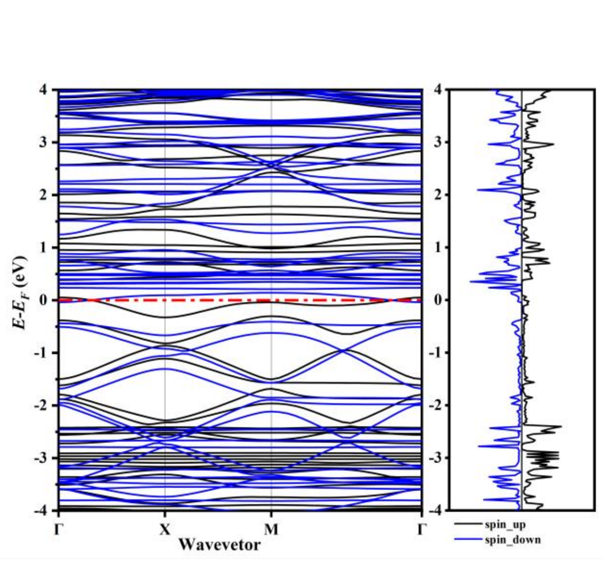

Figure S6. The band structure and the total density of states of B_2Mn_2. The Fermi level is assigned at zero.

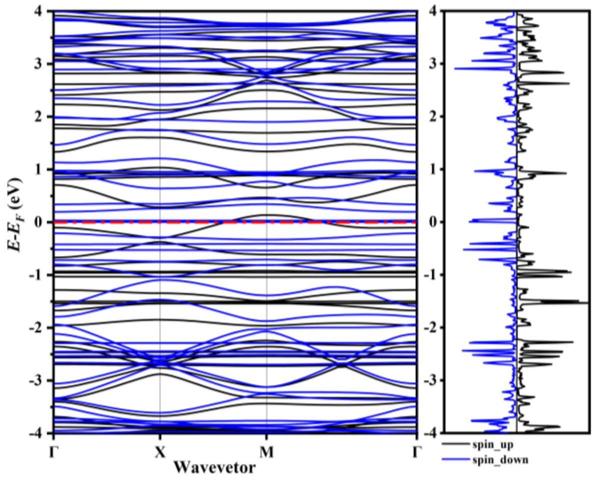

Figure S7. The band structure and the total density of states of B_2Os_2. The Fermi level is assigned at zero.

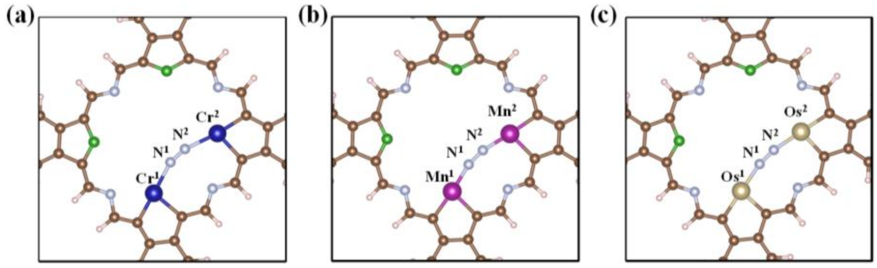

Figure S8. Top view of N_2 side-on adsorption on (a) B_2Cr_2, (b) B_2Mn_2, and (c) B_2Os_2.

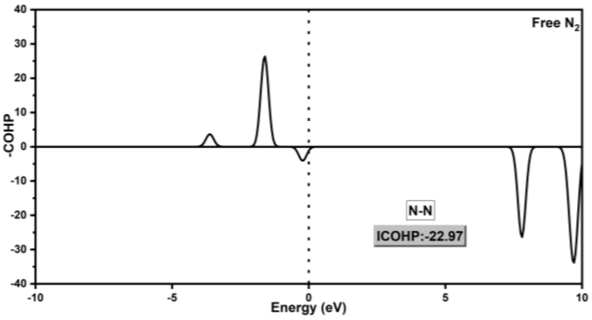

Figure S9. The COHP of free N_2.

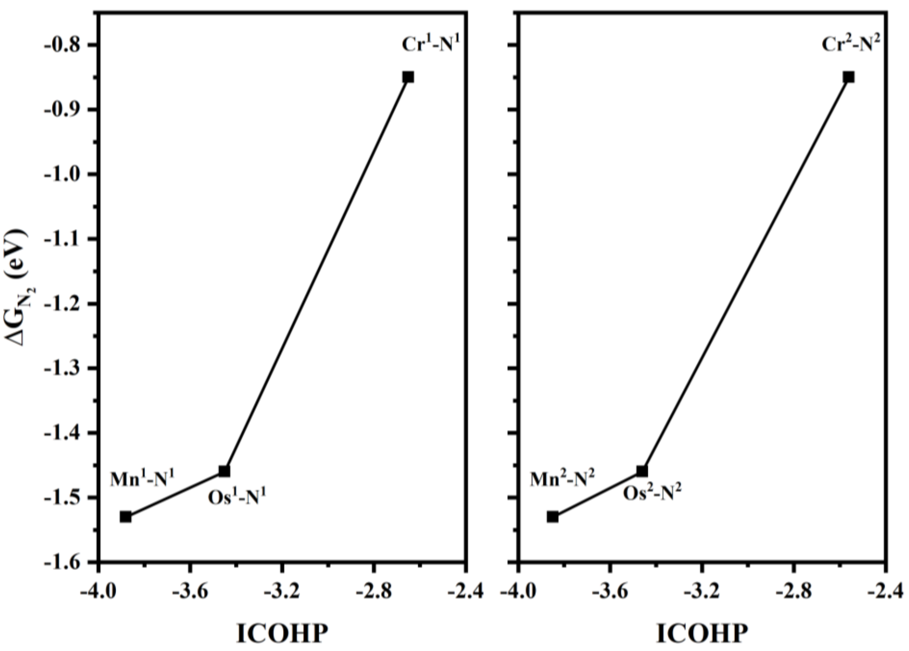

Figure S10. ICOHP of the bonding between N_2 and each metal, and the corresponding N_2 adsorption energy on bimetallic sites.

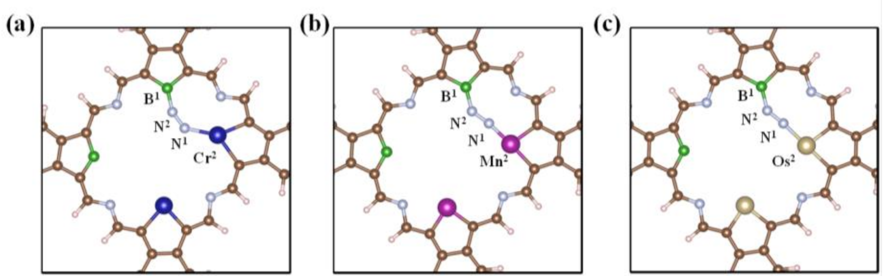

Figure S11. Top view of N_2 adsorption between B and metal on (a) B_2Cr_2, (b) B_2Mn_2, and (c) B_2Os_2.

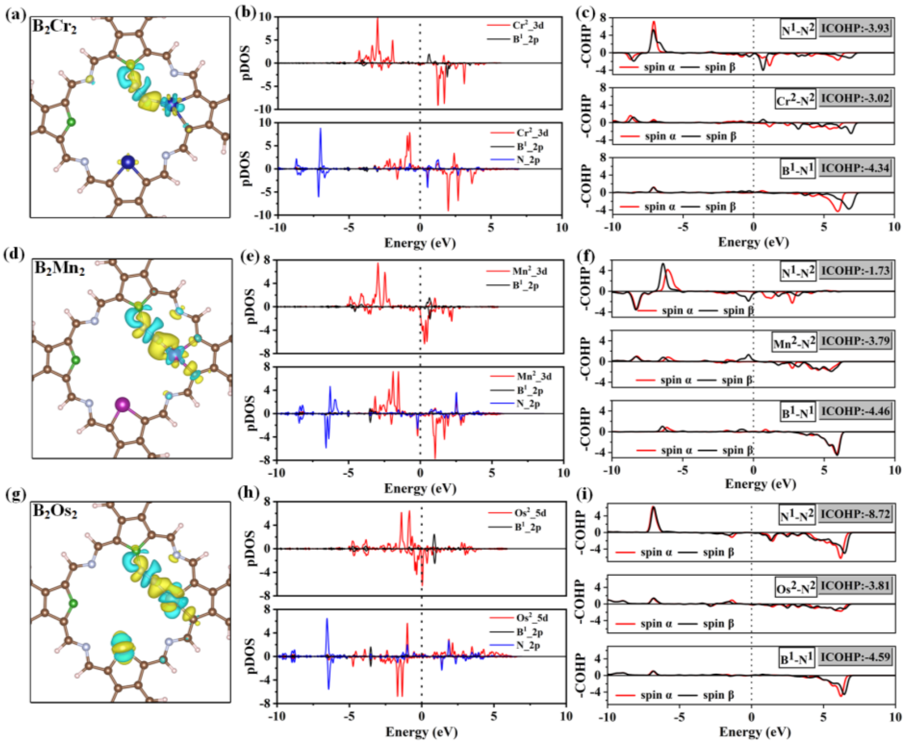

Figure S12. Charge difference density, pDOS and COHP of absorbed N_2 with side-on pattern on B and metal sites on (a-c) B_2Cr_2, (d-f) B_2Mn_2 and (g-i) B_2Os_2 catalysts. In the pDOS diagram, "N" represents the nitrogen atom of N_2. The contour level is set to 0.005 e/Å^3.

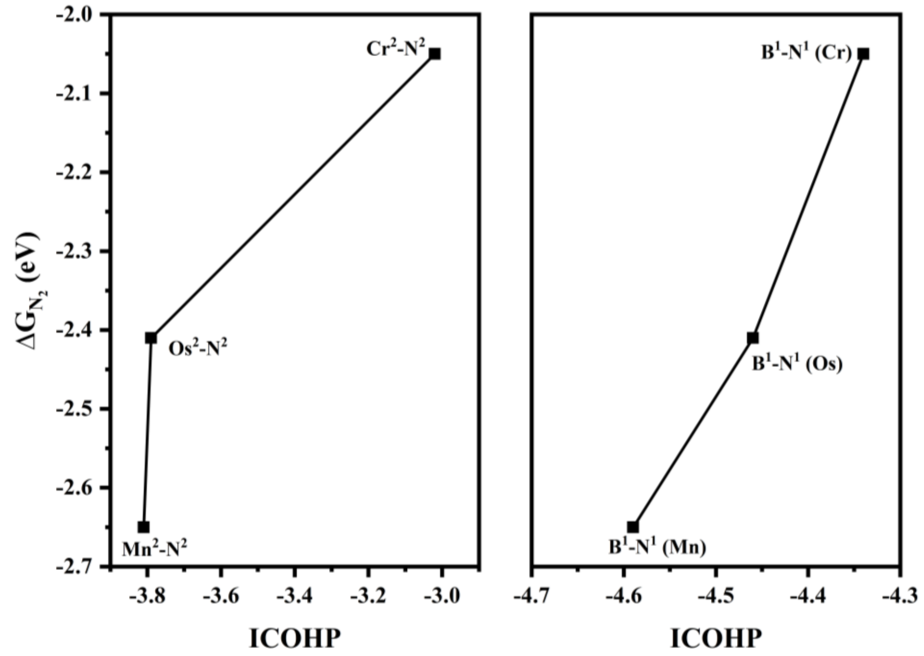

Figure S13. ICOHP and N_2 adsorption energy on B and metal sites for the bonding between metal and N (left) and between B and N (right).

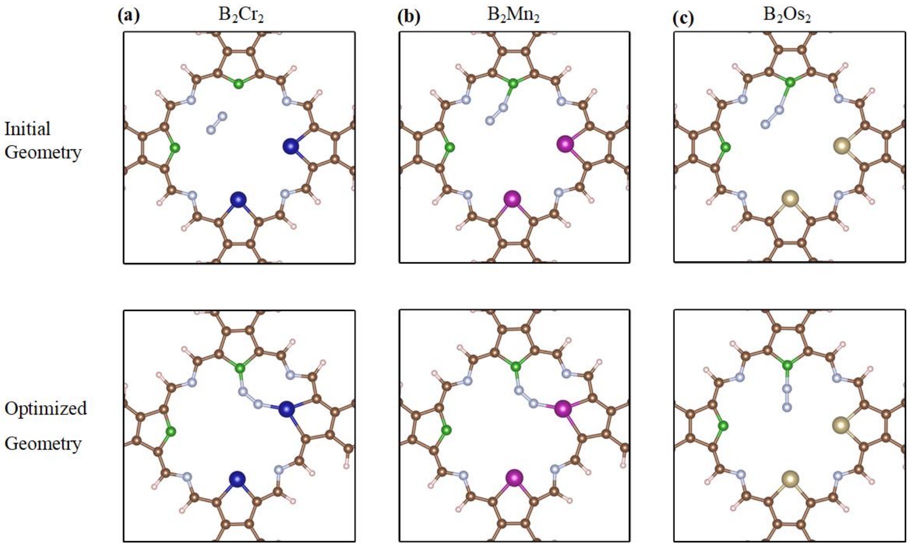

Figure S14. The initial and optimized top views of N_2 adsorption between B atoms on (a) B_2Cr_2, (b) B_2Mn_2, and (c) B_2Os_2.

Table S3. Free energy changes for each step in urea synthesis via the NCON mechanism on bimetallic sites on B_2Cr_2, B_2Mn_2 and B_2Os_2 catalysts.

System	Elementary steps	ΔG (eV)
B_2Cr_2	* + N_2 → **N_2	-0.85
	**N_2 → *NN*	-1.82
	NN + CO → *NCON*	-2.42
	NCON + H^+ + e^- → *NCON*H	0.60
	*NCON*H + H^+ + e^- → H*NCON*H	1.09
	*NCON*H + H^+ + e^- → *NCON*H_2	1.49
	H*NCON*H + H^+ + e^- → H*NCON*H_2	0.17
	NCONH_2 + H^+ + e^- → H*NCON*H_2	-0.22
	H*NCON*H_2 + H^+ + e^- → H_2*NCON*H_2	1.21
B_2Mn_2	* + N_2 → **N_2	-1.53
	**N_2 → *NN*	-1.06
	NN + CO → *NCON*	-0.21
	NCON + H^+ + e^- → *NCON*H	-0.06
	*NCON*H + H^+ + e^- → H*NCON*H	0.93
	*NCON*H + H^+ + e^- → *NCON*H_2	0.63
	H*NCON*H + H^+ + e^- → H*NCON*H_2	-0.64
	NCONH_2 + H^+ + e^- → H*NCON*H_2	-0.34
	H*NCON*H_2 + H^+ + e^- → H_2*NCON*H_2	0.35
B_2Os_2	* + N_2 → **N_2	-1.46
	**N_2 + CO → *NCON*	-1.15
	NCON + H^+ + e^- → *NCON*H	0.46
	*NCON*H + H^+ + e^- → H*NCON*H	-0.92
	*NCON*H + H^+ + e^- → *NCON*H_2	-1.24
	H*NCON*H + H^+ + e^- → H*NCON*H_2	0.22
	NCONH_2 + H^+ + e^- → H*NCON*H_2	0.53
	H*NCON*H_2 + H^+ + e^- → H_2*NCON*H_2	-0.16

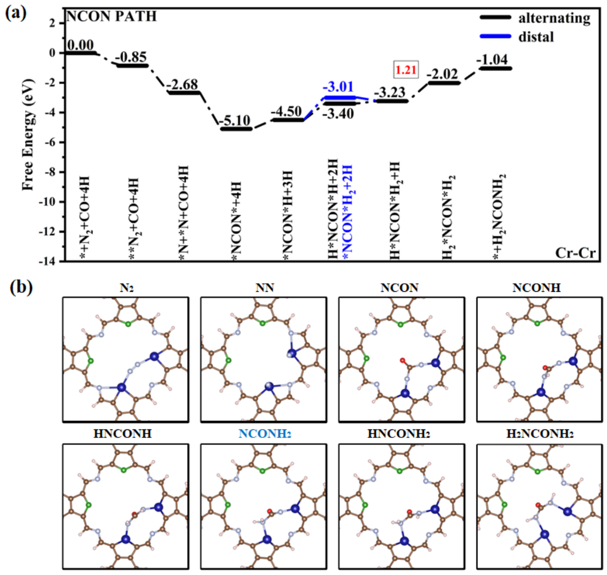

Figure S15. (a) Pathway diagram for urea synthesis via the NCON mechanism on Cr and Cr sites of B_2Cr_2 catalyst and (b) intermediate configurations.

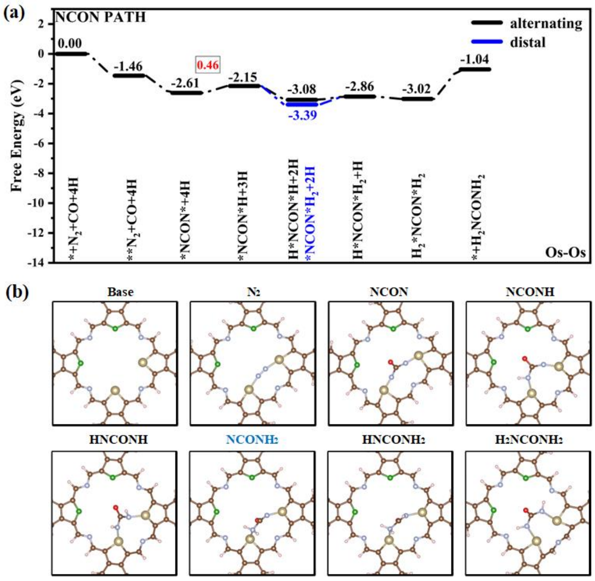

Figure S16. (a) Pathway diagram for urea synthesis via the NCON mechanism between Os and Os sites of B_2Os_2 system and (b) intermediate configurations.

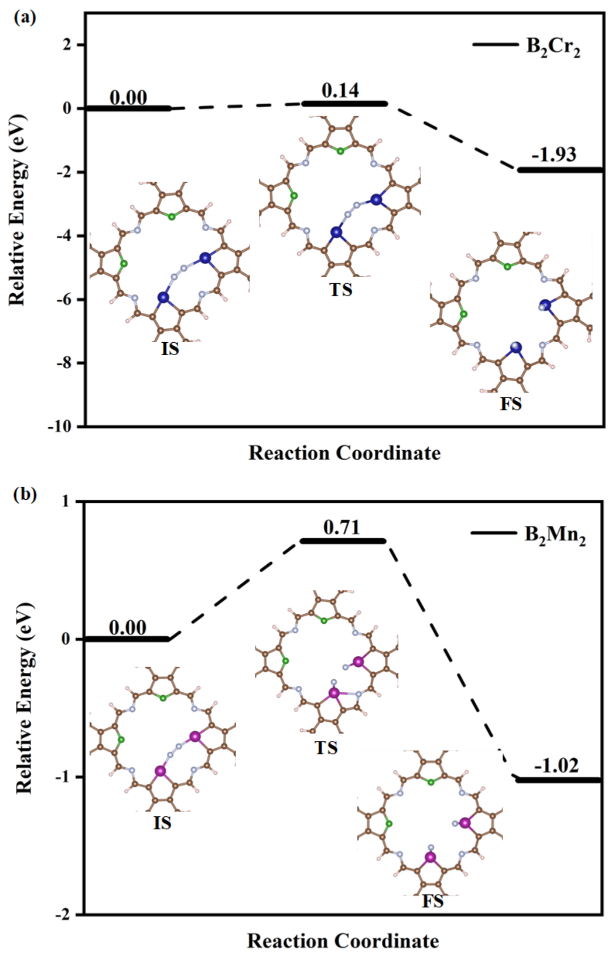

Figure S17.The computed reaction pathway for the reaction of N≡N bond dissociation on bimetallic sites on (a)B_2Cr_2 and (b)B_2Mn_2 catalysts.

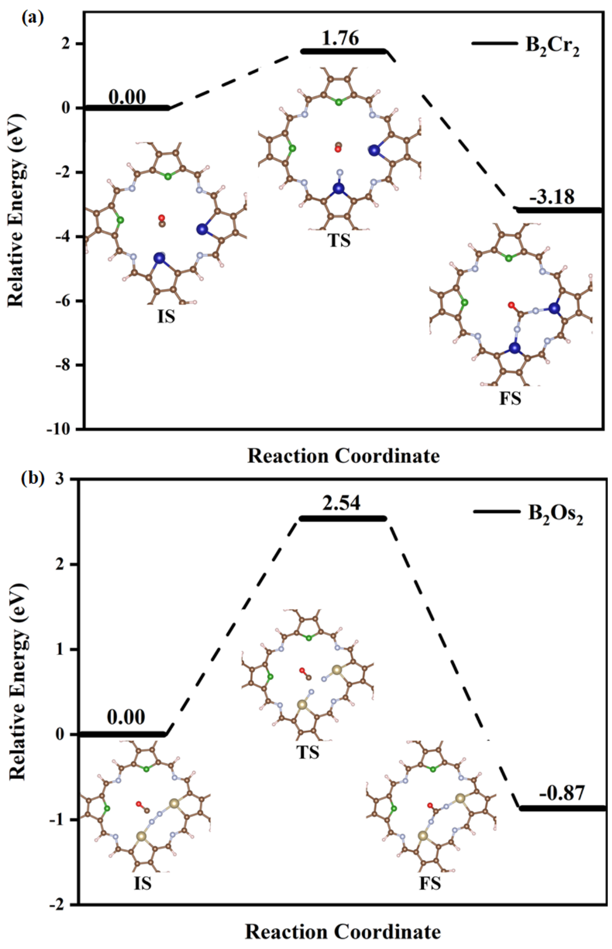

Figure S18.The computed reaction pathway for the C–N coupling of CO and $^*N_2$ on bimetallic sites on (a)B_2Cr_2 and (b)B_2Os_2 catalysts.

Table S4. Free energy changes for each step in urea synthesis via the CO mechanism on bimetal sites on B_2Cr_2, B_2Mn_2 and B_2Os_2 catalysts.

System	Elementary steps	ΔG (eV)
B_2Cr_2	$* + N_2 \rightarrow **N_2$	-0.85
	$**N_2 \rightarrow *NN*$	-1.82
	$*NN* + H^+ + e^- \rightarrow *NN*H$	0.10
	$*NN*H + H^+ + e^- \rightarrow *NN*H_2$	-0.25
	$*NN*H + H^+ + e^- \rightarrow H*NN*H$	0.28
	$H*NN*H + H^+ + e^- \rightarrow H*NN*H_2$	0.03
	$*NN*H_2 + H^+ + e^- \rightarrow H*NN*H_2$	0.56
	$H*NN*H_2 + H^+ + e^- \rightarrow H_2*NN*H_2$	-0.58
	$H_2*NN*H_2 + CO \rightarrow H_2*NCON*H_2$	0.83
B_2Mn_2	$* + N_2 \rightarrow **N_2$	-1.53
	$**N_2 \rightarrow *NN*$	-1.06
	$*NN* + H^+ + e^- \rightarrow *NN*H$	0.60
	$*NN*H + H^+ + e^- \rightarrow H*NN*H$	0.22
	$*NN*H + H^+ + e^- \rightarrow *NN*H_2$	0.08
	$H*NN*H + H^+ + e^- \rightarrow H*NN*H_2$	-0.01
	$*NN*H_2 + H^+ + e^- \rightarrow H*NN*H_2$	0.13
	$H*NN*H_2 + H^+ + e^- \rightarrow H_2*NN*H_2$	0.32
	$H_2*NN*H_2 + CO \rightarrow H_2*NCON*H_2$	-0.77
B_2Os_2	$* + N_2 \rightarrow **N_2$	-1.46
	$**N_2 + H^+ + e^- \rightarrow *NN*H$	0.05
	$*NN*H + H^+ + e^- \rightarrow H*NN*H$	0.42
	$*NN*H + H^+ + e^- \rightarrow *NN*H_2$	0.79
	$H*NN*H + H^+ + e^- \rightarrow H*NN*H_2$	-0.30
	$*NN*H_2 + H^+ + e^- \rightarrow H*NN*H_2$	-0.68
	$H*NN*H_2 + H^+ + e^- \rightarrow H_2*NN*H_2$	-1.05
	$H_2*NN*H_2 + CO \rightarrow H_2*NCON*H_2$	-0.66

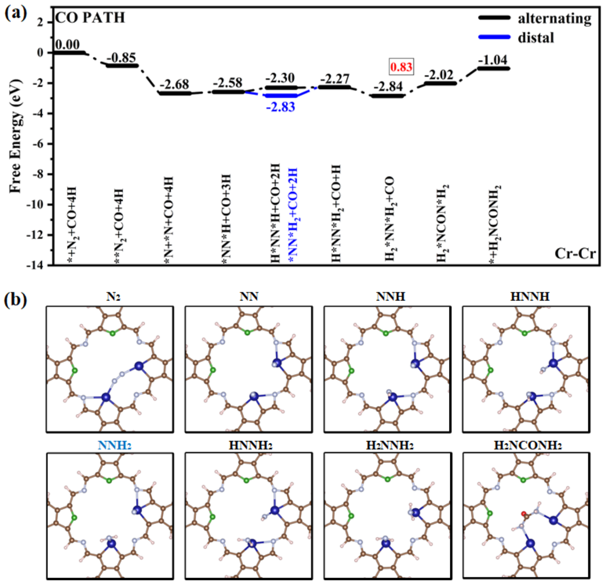

Figure S19. (a) Pathway diagram for urea synthesis via the CO mechanism on Cr and Cr sites of B_2Cr_2 catalyst and (b) intermediate configurations.

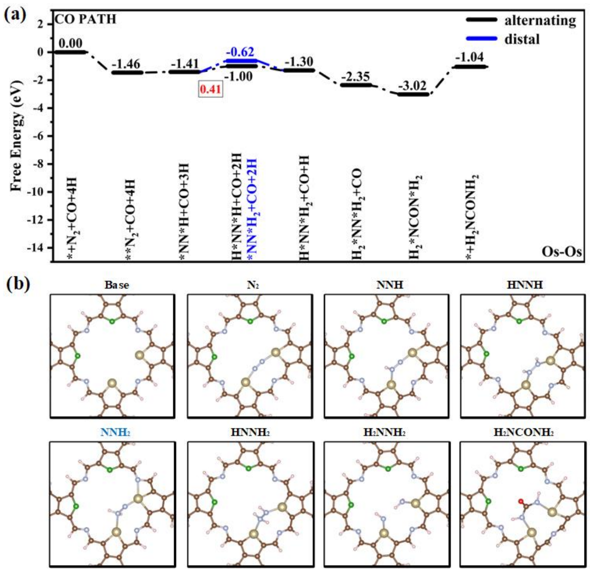

Figure S20. (a) Pathway diagram for urea synthesis via the CO mechanism on Os and Os sites of B_2Os_2 catalyst and (b) intermediate configurations.

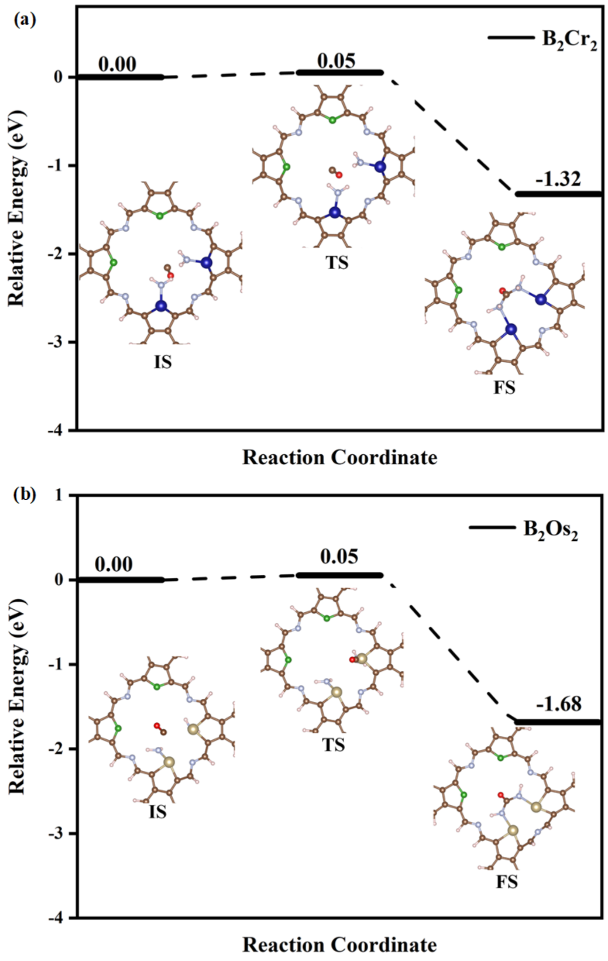

Figure S21. The computed reaction pathway for the C–N coupling of CO and H_2*NN*H_2 on bimetallic sites on (a) B_2Cr_2 and (b) B_2Os_2 catalysts.

Table S5. Free energy change for each step in urea synthesis via the NCON mechanism on B and metal sites on B_2Cr_2, B_2Mn_2 and B_2Os_2 catalysts. The black represents the N atom on the B side that is preferentially hydrogenated, while the green represents the N atom on the metal side that is preferentially hydrogenated.

Elementary steps	B_2Cr_2 ΔG (eV)	B_2Mn_2 ΔG (eV)	B_2Os_2 ΔG (eV)
* + N_2 → **N_2	-2.05	-2.41	-2.65
**N_2 + CO → *NCON*	-1.76	0.27	0.49
NCON + H^+ + e^- → *NCON*H	-0.74	-1.67	-1.56
*NCON*H + H^+ + e^- →H*NCON*H	-0.69	-0.22	-0.49
*NCON*H + H^+ + e^- → *NCON*H_2	0.82	0.44	0.28
H*NCON*H + H^+ + e^- →H*NCON*H_2	1.37	0.34	-0.09
NCONH_2 + H^+ + e^- →H*NCON*H_2	-0.14	-0.32	-0.86
H*NCON*H_2 + H^+ + e^- →H_2*NCON*H_2	0.85	-0.12	0.99
NCON + H^+ + e^- →H*NCON*	0.52	-0.26	-1.89
H*NCON* + H^+ + e^- →H*NCON*H	-1.94	-1.63	-0.16
H*NCON* + H^+ + e^- →H_2*NCON*	-1.54	-2.95	-0.22
H*NCON*H + H^+ + e^- →H_2*NCON*H	0.92	-0.39	-0.27
H_2*NCON* + H^+ + e^- →H_2*NCON*H	0.52	0.93	-0.20
H_2*NCON*H + H^+ + e^- →H_2*NCON*H_2	1.30	0.61	1.17

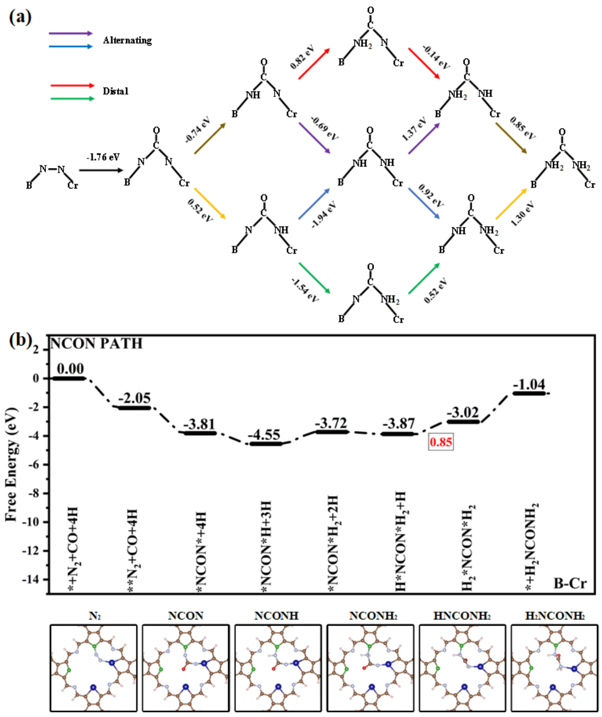

Figure S22. (a) The diagram of free energy changes for urea synthesis via the NCON mechanism on B and Cr sites on B_2Cr_2 catalyst. The connecting lines between elements only represent bonding, and do not indicate bond order. The brown and yellow paths represent adding H to the N atom on the boron side and adding H to the N atom on the metal side, respectively. The purple path represents the alternating mechanism of adding H to the N atom on the B side, the blue path represents the alternating mechanism of adding hydrogen to the N atom on the metal side, the red path represents the distal mechanism of adding hydrogen to the N atom on the B side, and the green path represents the distal mechanism of adding hydrogen to the N atom on the metal side. (b) One of the optimal pathways for urea synthesis via the NCON mechanism on B and Cr sites on B_2Cr_2 catalyst.

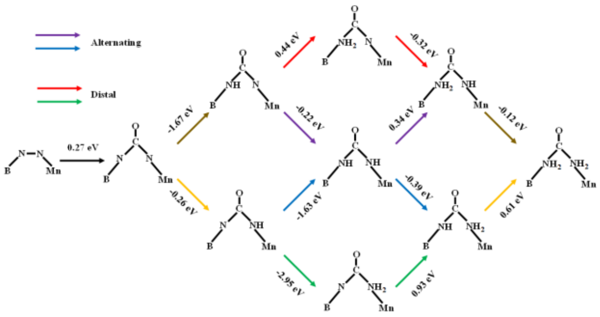

Figure S23. The diagram of free energy changes for urea synthesis via the NCON mechanism on B and Mn sites on B_2Mn_2 catalyst. The connecting lines between elements only represent bonding, and do not indicate bond order. The brown and yellow paths represent adding H to the N atom on the boron side and adding H to the N atom on the metal side, respectively. The purple path represents the alternating mechanism of adding H to the N atom on the B side, the blue path represents the alternating mechanism of adding hydrogen to the N atom on the metal side, the red path represents the distal mechanism of adding hydrogen to the N atom on the B side, and the green path represents the distal mechanism of adding hydrogen to the N atom on the metal side.

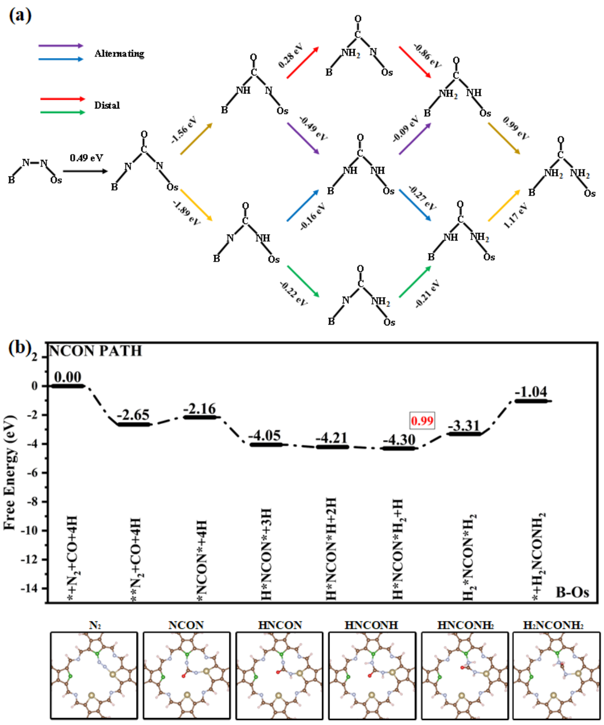

Figure S24. (a) The diagram of free energy changes for urea synthesis via the NCON mechanism on B and Os sites on B_2Os_2 catalyst. The connecting lines between elements only represent bonding, and do not indicate bond order. The brown and yellow paths represent adding H to the N atom on the boron side and adding H to the N atom on the metal side, respectively. The purple path represents the alternating mechanism of adding H to the N atom on the B side, the blue path represents the alternating mechanism of adding hydrogen to the N atom on the metal side, the red path represents the distal mechanism of adding hydrogen to the N atom on the B side, and the green path represents the distal mechanism of adding hydrogen to the N atom on the metal side. (b) One of the optimal pathways for urea synthesis via the NCON mechanism on B and Os sites on B_2Os_2 catalyst.

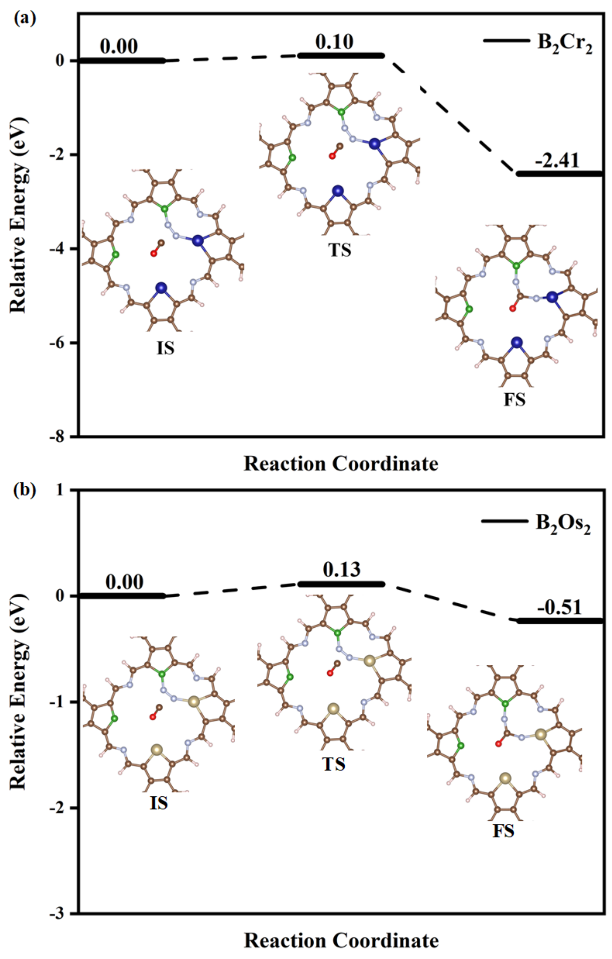

Figure S25. The computed reaction pathway for the C–N coupling of CO and $*N_2$ on B and metal sites on B_2Cr_2 and B_2Os_2 catalysts.

Table S6. Free energy change for each step in urea synthesis via the CO mechanism on B and metal sites on B_2Cr_2, B_2Mn_2, and B_2Os_2 catalysts. The black represents the N atom on the B side that is preferentially hydrogenated, while the green represents the N atom on the metal side that is preferentially hydrogenated.

Elementary steps	B_2Cr_2 ΔG (eV)	B_2Mn_2 ΔG (eV)	B_2Os_2 ΔG (eV)
* + N_2 → **N_2	-2.05	-2.41	-2.65
**N_2 + H^+ + e^- → *NN*H	-0.93	0.45	-0.33
*NN*H + H^+ + e^- → H*NN*H	0.48	-0.29	0.62
*NN*H + H^+ + e^- → *NN*H_2	0.82	0.93	1.65
H*NN*H + H^+ + e^- → H*NN*H_2	0.53	0.65	-1.54
NNH_2 + H^+ + e^- →H*NNH_2	0.19	-0.58	-2.56
H*NN*H_2 + H^+ + e^- →H_2*NN*H_2	-2.82	-0.24	-0.39
H_2*NN*H_2 + CO →H_2*NCON*H_2	1.79	-1.97	0.97
**N_2 + H^+ + e^- → H*NN*	0.04	0.89	1.11
H*NN* + H^+ + e^- →H*NN*H	-0.50	-0.74	-0.82
H*NN* + H^+ + e^- →H_2*NN*	0.39	0.16	0.46
H*NN*H + H^+ + e^- →H_2*NN*H	0.20	-0.20	0.04
H_2*NN* + H^+ + e^- →H_2*NN*H	-0.68	-1.09	-1.25
H_2*NN*H + H^+ + e^- →H_2*NN*H_2	-2.49	0.61	-1.96

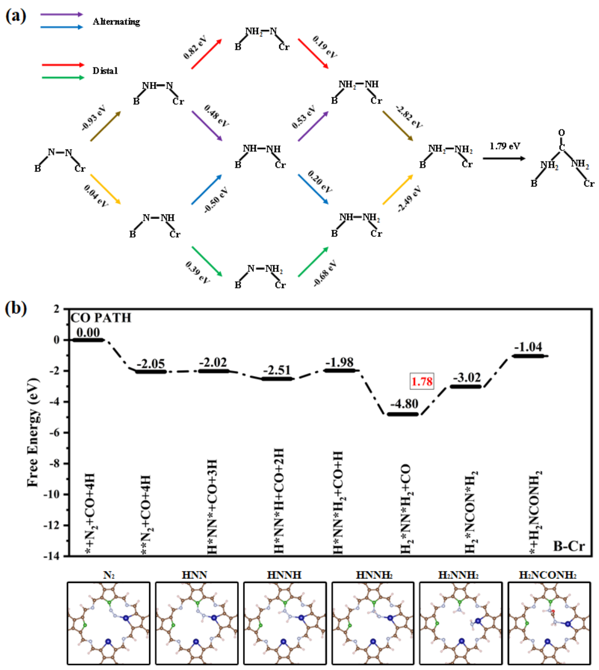

Figure S26. (a) The diagram of free energy changes for urea synthesis via the CO mechanism on B and Cr sites on B_2Cr_2 catalyst. The connecting lines between elements only represent bonding, and do not indicate bond order. The brown and yellow paths represent adding H to the N atom on the boron side and adding H to the N atom on the metal side, respectively. The purple path represents the alternating mechanism of adding H to the N atom on the B side, the blue path represents the alternating mechanism of adding hydrogen to the N atom on the metal side, the red path represents the distal mechanism of adding hydrogen to the N atom on the B side, and the green path represents the distal mechanism of adding hydrogen to the N atom on the metal side. (b) One of the optimal pathways for urea synthesis via the CO mechanism on B and Cr sites on B_2Cr_2 catalyst.

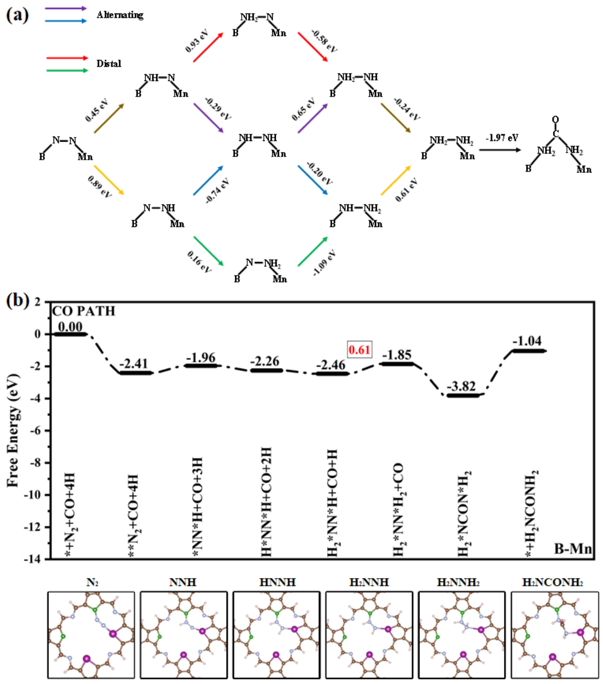

Figure S27. (a) The diagram of free energy changes for urea synthesis via the CO mechanism on B and Mn sites on B_2Mn_2 catalyst. The connecting lines between elements only represent bonding, and do not indicate bond order. The brown and yellow paths represent adding H to the N atom on the boron side and adding H to the N atom on the metal side, respectively. The purple path represents the alternating mechanism of adding H to the N atom on the B side, the blue path represents the alternating mechanism of adding hydrogen to the N atom on the metal side, the red path represents the distal mechanism of adding hydrogen to the N atom on the B side, and the green path represents the distal mechanism of adding hydrogen to the N atom on the metal side. (b) One of the optimal pathways for urea synthesis via the CO mechanism on B and Mn sites on B_2Mn_2 catalyst.

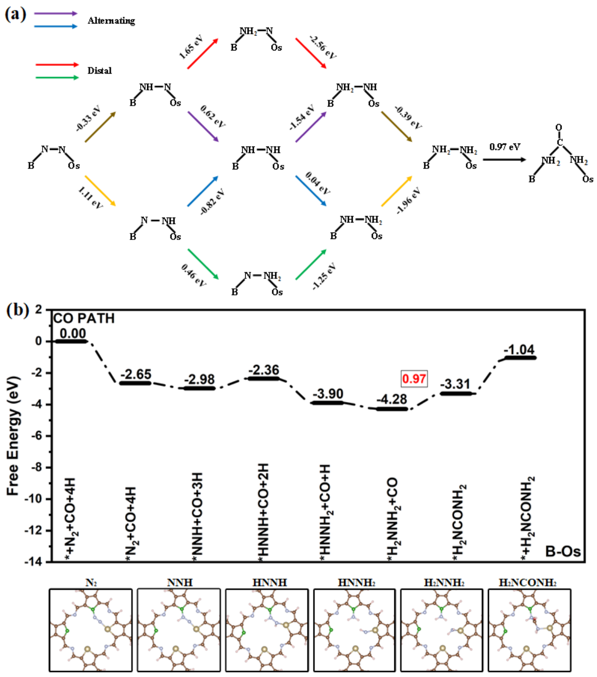

Figure S28. (a) The diagram of free energy changes for urea synthesis via CO mechanism on B and Os sites on B_2Os_2 catalyst. The connecting lines between elements only represent bonding, and do not indicate bond order. The brown and yellow paths represent adding H to the N atom on the boron side and adding H to the N atom on the metal side, respectively. The purple path represents the alternating mechanism of adding H to the N atom on the B side, the blue path represents the alternating mechanism of adding hydrogen to the N atom on the metal side, the red path represents the distal mechanism of adding hydrogen to the N atom on the B side, and the green path represents the distal mechanism of adding hydrogen to the N atom on the metal side. (b) One of the optimal pathways for urea synthesis via the CO mechanism on B and Os sites on B_2Os_2 catalyst.

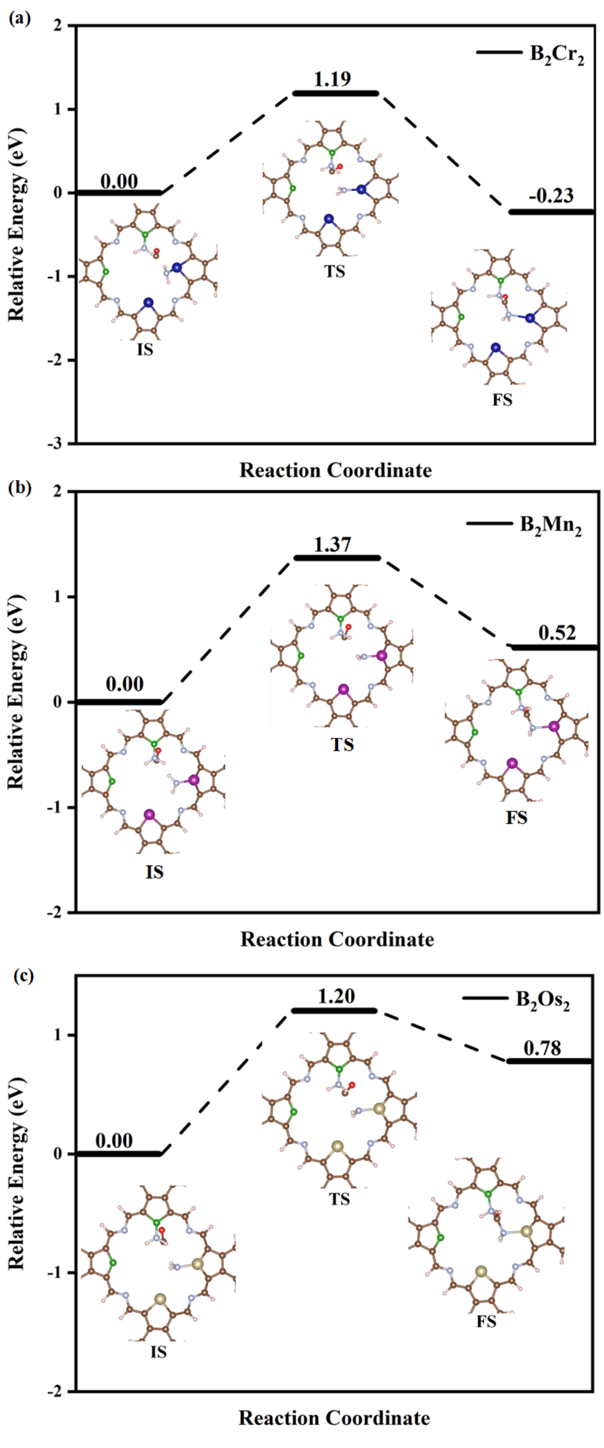

Figure S29. The computed reaction pathway for the C–N coupling of CO and H_2*NN*H_2 on B and metal sites on (a) B_2Cr_2 and (b) B_2Mn_2 and (c) B_2Os_2 catalysts.

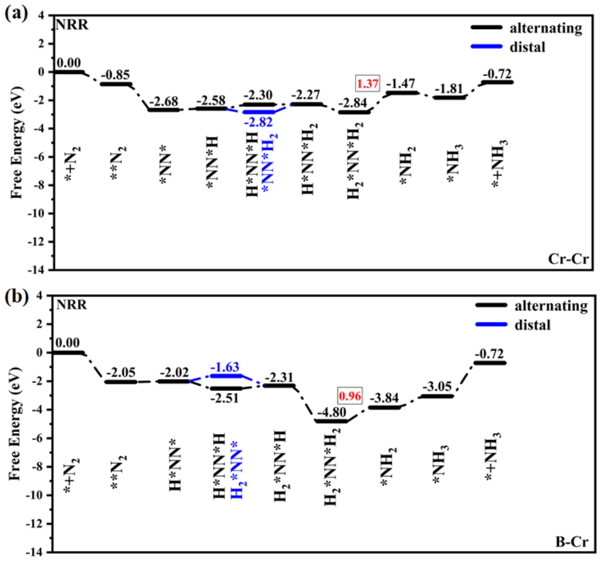

Figure S30. (a) Free energy change diagram for NRR on Cr and Cr sites on B_2Cr_2 catalyst. (b) Free energy change diagram for NRR on B and Cr sites on B_2Cr_2 catalyst.

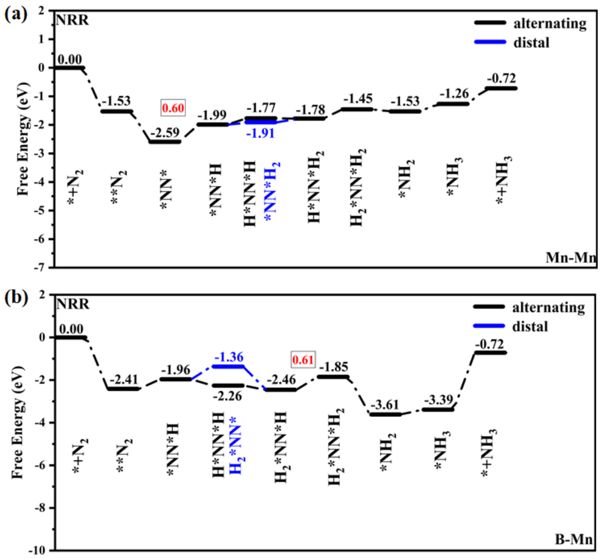

Figure S31. (a) Free energy change diagram for NRR on Mn and Mn sites on B_2Mn_2 catalyst. (b) Free energy change diagram for NRR on B and Mn sites on B_2Mn_2 catalyst.

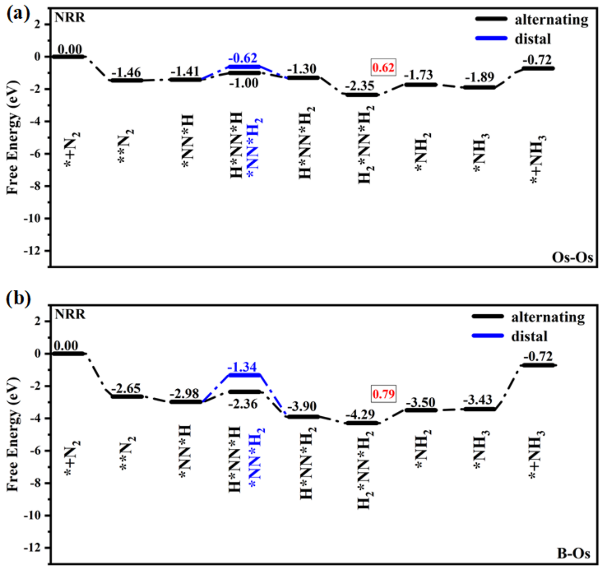

Figure S32. (a) Free energy change diagram for NRR on Os and Os sites on B_2Os_2 catalyst. (b) Free energy change diagram for NRR on B and Os sites on B_2Os_2 catalyst.

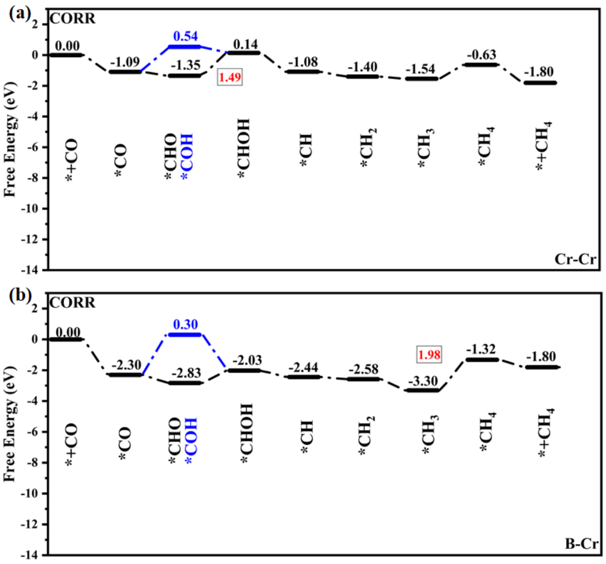

Figure S33. (a) Free energy change diagram for CORR on Cr and Cr sites on B_2Cr_2 catalyst. (b) Free energy change diagram for CORR on B and Cr sites on B_2Cr_2 catalyst.

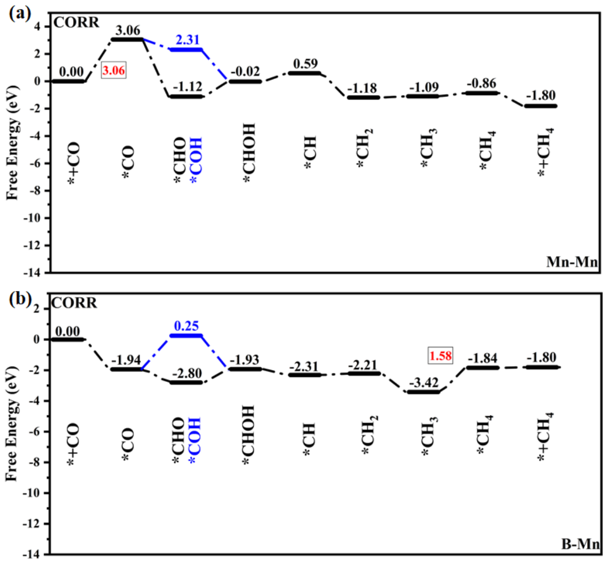

Figure S34. (a) Free energy change diagram for CORR on Mn and Mn sites on B_2Mn_2 catalyst. (b) Free energy change diagram for CORR on B and Mn sites on B_2Mn_2 catalyst.

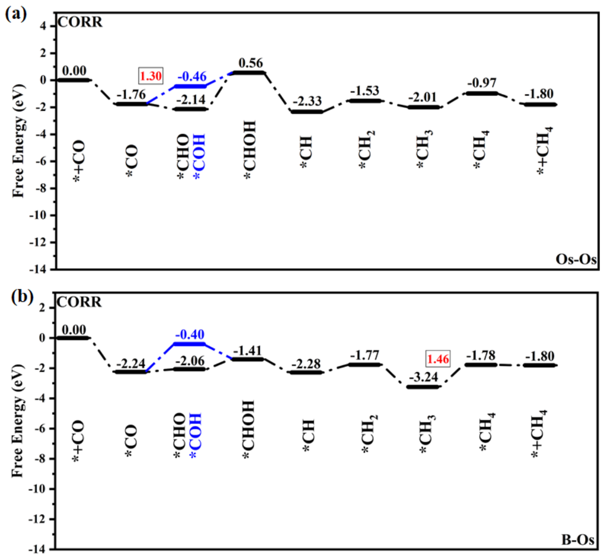

Figure S35. (a) Free energy change diagram for CORR on Os and Os sites on B_2Os_2 catalyst. (b) Free energy change diagram for CORR on B and Os sites on B_2Os_2 catalyst.
